# Combining nitric oxide release with anti-inflammatory activity preserves nigrostriatal dopaminergic innervation and prevents motor impairment in a 1-methyl-4-phenyl-1,2,3,6-tetrahydropyridine model of Parkinson's disease

**DOI:** 10.1186/1742-2094-7-83

**Published:** 2010-11-23

**Authors:** Francesca L'Episcopo, Cataldo Tirolo, Salvatore Caniglia, Nunzio Testa, Pier A Serra, Francesco Impagnatiello, Maria C Morale, Bianca Marchetti

**Affiliations:** 1 OASI Institute for Research and Care on Mental Retardation and Brain Aging (IRCCS), Neuropharmacology Section, 94018 Troina, Italy; 2 Department of Pharmacology, Faculty of Medicine, University of Sassari, 07100 Sassari, Italy; 3 Nicox Research Institute, Bresso, Milan, Italy; 4 Department of Clinical and Molecular Biomedicine, Pharmacology Section, Faculty of Medicine, University of Catania, 95125 Catania, Italy; 5 Faculty of Pharmacy, University of Catania, 95125 Catania, Italy

## Abstract

**Background:**

Current evidence suggests a role of neuroinflammation in the pathogenesis of Parkinson's disease (PD) and in the 1-methyl-4-phenyl-1,2,3,6-tetrahydropyridine (MPTP) model of basal ganglia injury. Reportedly, nonsteroidal anti-inflammatory drugs (NSAIDs) mitigate DAergic neurotoxicity in rodent models of PD. Consistent with these findings, epidemiological analysis indicated that certain NSAIDs may prevent or delay the progression of PD. However, a serious impediment of chronic NSAID therapy, particularly in the elderly, is gastric, renal and cardiac toxicity. Nitric oxide (NO)-donating NSAIDs, have a safer profile while maintaining anti-inflammatory activity of parent compounds. We have investigated the oral activity of the NO-donating derivative of flurbiprofen, [2-fluoro-α-methyl (1,1'-biphenyl)-4-acetic-4-(nitrooxy)butyl ester], HCT1026 (30 mg kg^-1 ^daily in rodent chow) in mice exposed to the parkinsonian neurotoxin MPTP.

**Methods:**

Ageing mice were fed with a control, flurbiprofen, or HCT1026 diet starting ten days before MPTP administration and continuing for all the experimental period. Striatal high affinity synaptosomial dopamine up-take, motor coordination assessed with the rotarod, tyrosine hydroxylase (TH)- and dopamine transporter (DAT) fiber staining, stereological cell counts, immunoblotting and gene expression analyses were used to assess MPTP-induced nigrostriatal DAergic toxicity and glial activation 1-40 days post-MPTP.

**Results:**

HCT1026 was well tolerated and did not cause any measurable toxic effect, whereas flurbiprofen fed mice showed severe gastrointestinal side-effects. HCT1026 efficiently counteracted motor impairment and reversed MPTP-induced decreased synaptosomal [^3^H]dopamine uptake, TH- and DAT-stained fibers in striatum and TH^+ ^neuron loss in subtantia nigra pars compacta (SNpc), as opposed to age-matched mice fed with a control diet. These effects were associated to a significant decrease in reactive macrophage antigen-1 (Mac-1)-positive microglial cells within the striatum and ventral midbrain, decreased expression of iNOS, Mac-1 and NADPH oxidase (PHOX), and downregulation of 3-Nitrotyrosine, a peroxynitrite finger print, in SNpc DAergic neurons.

**Conclusions:**

Oral treatment with HCT1026 has a safe profile and a significant efficacy in counteracting MPTP-induced dopaminergic (DAergic) neurotoxicity, motor impairment and microglia activation in ageing mice. HCT1026 provides a novel promising approach towards the development of effective pharmacological neuroprotective strategies against PD.

## Background

Selective degeneration of dopaminergic (DAergic) neurons in the subtantia nigra pars compacta (SN) is a pathological hallmark of both Parkinson's disease (PD) and 1-methyl-4-phenyl-1,2,3,6-tetrahydropyridine (MPTP) animal model of PD. The decline of dopamine in the striatum is associated clinically with progressive bradykinesia, tremor, rigidity and postural instability [1]. Current DAergic treatments improve the motor symptoms and quality of life for patients during the early stages of PD but do not prevent the progression of the disease associated with disabling side-effects [2]. With the exception of inherited cases linked to specific gene defects that account for <10% of cases, PD is a sporadic condition of unknown causes. Besides host genetics, environment, age, gender and inflammatory processes are factors affecting disease onset and/or progression [3-18].

Activation of microglia, a hallmark of neuroinflammation, has been demonstrated in the SN of PD patients [19], in human patients exposed to MPTP [20], and in experimental models of PD [21-35]. Accumulation of reactive oxygen species (ROS), inflammatory-associated factors including cycloxygenase-2 (COX-2) and inducible-nitric oxide synthase (iNOS)-derived NO, and pro-inflammatory cytokines (including TNF-α, IL-1β and IFN-γ) in the SN of PD patients further support that a state of chronic inflammation characterizes PD brain [5,22-26]. In addition, elevated expression of macrophage-antigen complex 1 (Mac-1), a β_2_-integrin family member expressed exclusively in microglia, and NADPH oxidase (PHOX), one of the major sources for production of ROS or related reactive nitric species (RNS) in activated microglia, have been reported in PD animal models [8,11,13-16,22-35]. In keeping with these findings, genetic or pharmacological inhibition of most inflammatory factors, including iNOS, PHOX, Mac-1 and COX-2-derived mediators, significantly attenuated DAergic degeneration in experimental models of PD [27-44]. Conversely, blocking the action of endogenous anti-inflammatory molecules, such as glucocorticoid hormones in transgenic mice expressing a glucocorticoid receptor (GR) antisense RNA, sharply increases microglial activation in response to MPTP, resulting in increased DAergic neuron vulnerability [8,10,11].

Consistent with the inflammation hypothesis, epidemiological analysis has indicated that nonsteroidal anti-inflammatory drugs (NSAIDs) may prevent or delay the progression of PD [6,7,45-52]. NSAIDs are among the most widely used therapeutic agents for the treatment of pain, fever and inflammation. Their effects are largely attributed to the inhibition of the enzymatic activity of COXs, of which there are two isoforms, COX-1 and COX-2. Both enzymes are responsible for arachidonic acid conversion in different prostaglandins (PGs) [53,54]. While COX-1 is constitutively expressed in most tissues, COX-2 is induced during pathophyiological responses to inflammatory stimuli [55]. Both mixed and selective COX-2 inhibitors have been reported to mitigate DAergic neurotoxicity in experimental models of PD; or to reduce LPS-induced neuronal damage [recently reviewed in [45,46]]. Besides targeting COXs, NSAIDs can act in a COX-independent way, which includes activation of the nuclear factor peroxisome proliferator-activated receptor-γ (PPAR-γ), the protection against glutamate and 1-methyl-4-phenylpyrdinium ion (MPP^+^) toxicity, scavenging hydroxyl and NO radicals and dopamine-quinone formation [18,45-48].

Nevertheless, the long-term therapy with non-selective NSAIDs is characterized by significant adverse effects on gastrointestinal tract and kidneys, whereas increased risk of cardiovascular events has been reported with COX-2-selective inhibitors [56], which may limit their clinical use in chronic conditions. The nitric oxide (NO)-NSAID HCT1026 [2-fluoro-α-methyl(1,1'-biphenyl)-4-acetic-4-(nitrooxy)butyl ester], NO-donating flurbiprofen, belongs to a novel class of anti-inflammatory agents obtained by derivatization of conventional NSAIDs with a NO-donating moiety which strongly reduce their untoward side effects without altering the anti-inflammatory effectiveness [57-66].

We herein report that HCT1026 has a safer profile and a greater efficacy than its parent compound in rescuing nigrostriatal DAergic neurons from MPTP neurotoxicity and that a shift in microglial pro-inflammatory phenotype is involved in this phenomenon. HCT1026 is safe at the gastrointestinal level, and it has been tested in humans; it is effective on oral administration, and it is thus suited for long-term treatment, thereby representing a promising approach towards the development of effective pharmacological neuroprotective strategies against PD.

## Methods

### Animals

Young adult (2-5 months of age) and ageing (9-11 month-old) male C57BL/6 (Charles River, Calco, Italy) housed (5 mice/cage) in a temperature (21-23°C), humidity (60%), and light (50/50 light:dark cycle, lights on at 06.00 a.m) controlled room, with controlled access to food and water, were allowed to acclimate one week before the start of the experimental protocol. Studies were conducted in strict accord with the Guide for the Care and Use of Laboratory Animals (NIH), and approved by the Review Boards of the OASI Institute (Troina, Italy). The authors further attest that all efforts were made to minimize the number of animal used and their suffering.

### Drug administration

The drugs were compounded in the chow (Teklad 2018 diet, Harlan), the schedule of administration defined according to a pilot experiment conducted to monitor daily food intake, and the dose selected as that producing a full anti-inflammatory effect [66]. The following doses were used: HCT1026 190 ppm in the diet or 30 mg kg^-1 ^day^-1 ^per animal; flurbiprofen 120 ppm or 20 mg kg^-1 ^day^-1 ^per animal Flurbiprofen dose was equimolar to HCT1026 (MW HCT1026:361.4; flubiprofen:244.3, ratio HCT1026/flurbiprofen = 1.48). Plain teklas 2018 chow was used as control diet. The treatment started 7-10 d days prior MPTP administration and thoroughout the entire experiment. Food consumption was monitored daily, diets were weighed and and food intake calculated daily, body weights recorded.

### MPTP administration

Both the acute [67] and the subchronic [68] MPTP injection paradigms, and three different dose-levels (5, 15, or 30 mg kg^-1 ^MPTP-HCl measured as a free base), were selected in order to verify the ability of a preventive administration of HCT1026 to exert neuroprotective effects against MPTP-induced DAergic toxicity (Table 1). The same lot of MPTP-HCl (Sigma, Italy) was used for one experimental series. In a first series of experiments, in the acute protocol, MPTP was systemically injected (i.p.) at a dose of 15 mg/kg^-1^, 4 times a day, at 2 hr intervals [28,36]. In the subchronic regimen, increasing doses of MPTP were administrated i.p. at 24-h interval, for 5 consecutive days and mice sacrificed 7 d post-treatment. The dose of 15 mg/kg^-1 ^day^-1 ^and the subchronic regimen were then selected to assess longterm effects of HCT1026 in all subsequent experiments in ageing mice [32,42]. Groups of mice fed with the different diets and injected with vehicle (0.9% saline, 2 ml kg^-1 ^intraperitoneally), served as controls (see Table 1). MPTP handling and safety measures were in accordance with published guidelines according to Jackson-Lewis and Przedborski [69].

**Table 1:** Experimental design, animal number and analyses performed per time-point (tp) within each experimental group.

	Subacute MPTP						Subchronic MPTP						
**Days after MPTP discontinuation**													

**Analyses**	**mice/tp**	**-7**	**0**	**+1**	**+3**	**+7**	**0**	**+1**	**+3**	**+7**	**+21**	**+30**	**+40**

**Rotarod**	**10**	**+**	**+**	**+**	**+**	**+**							

**Neurochem**.	**5**		**+**			**+**		+		+	+	+	+

**Immunohistochem**.	**5**						+	+	+	+	+	+	+

**Microglia markers**	**5**						+	+	+	+			

**Gene expression**	**4**						+	+		+	+	+	+

**Western blot**	**4**						+	+	+	+	+		+

Young (3-5 month-old) and ageing (9-11 month-old) C57Bl/6 mice fed with a control, flurbiprofen or HCT1026 diets (30 mg kg^-1^) starting at -10 d, underwent an MPTP treatment according to the subacute (n = 4 intraperitoneal, i.p., injections of MPTP-HCl, 15 mg kg^-1 ^free base, 2 hours apart in one day) or subchronic (n = 5 intraperitoneal, i.p., injections of MPTP-HCl, at 5, 15 or 30 mg kg^-1 ^free base, every 24 h), injection paradigms. Age-matched mice fed with the different diets received physiologic saline (NaCl, 10 ml kg^-1^) and served as controls. Mice number and timepoints (tp) for each experimental protocol for each set of analyses/tp, are indicated. Striatum and ventral midbrains tissues were processed for RT-PCR or western blotting, as reported according to the different determinations. For immunohistochemical analyses, on the day of sacrifice mice were anesthetized and transcardially perfused, the brains processed as indicated. See Methods section for details.

### Sacrifice and tissue processing

Controls and MPTP-treated mice were killed at selected times ranging from 0-40 days post- MPTP treatment (dpt). To study early drug effects on microglia activation during the active degeneration phase, groups of mice were studied 1-7 dpt (Table 1). To monitor the severity of nigrostriatal damage and the survival/neurorescue of nigrostrial neurons, group of mice were studied 7, 21, 30 and 40 dpt. MPTP-induced motor deficit was assed with the Rotarod, at -7, + 1, + 3 and + 7 dpt. For neurochemical determinations, heads were cooled by rapid immersion in liquid nitrogen. Thereafter, striata of both sides and ventral mesencephalon, were rapidly removed and frozen at -80°C for subsequent determinations [8]. For histopathological determinations, mice were deeply anesthetized and perfused transcardially, as reported in full details [8].

### Determination of drug plasma level

Blood samples were taken at the indicated times and plasma samples were frozen and stored at -80° until the analysis was performed. Plasma 0.1 ml was mixed with 10 μl ketoprofen (internal standard, 1 mg ml^-1 ^stock solution in methanol) and 400 μl of cold methanol/acetonitrile (1:1) mixture, vortexed and centrifuged at 13,000 × g for 10 min at 25°C. HPLC analysis was performed on fixed volume of organic extraction mixture. Chromatographic analysis was performed on an Agilent 1100 series system equipped with a Diode array detector operating at 246-nm single wavelenghth. Separations were achieved with a gradient elution on a Synergi MAX-RP 80A column (150 × 2 mm i.d.; 4 μm) equipped with MAX-RP precolumn (4 × 2 mm i.d.). The mobile phase was acetonitrile and phosphoric acid 0.1%. The flow rate was 1 ml min^-1 ^and the column temperature was 25°C. Under these conditions, the retention time of HCT1026, flurbiprofen and internal standard were 5.7, 1.3, 0,8 min, respectively. Only flurbiprofen was detectable in plasma of HCT1026 treated mice. HCT1026 and its des-nitro metabolite (i.e. HCT1027) were undetectable in all plasma samples, as previously reported [70].

### Motor behavior analysis with the rotarod

An accelerating rotarod (five-lane accelerating rotarod; Ugo Basile, Comerio, Italy) was used to measure motor balance and coordination in mice. Mice have to keep their balance on a horizontal rotating rod (diameter, 3 cm) and rotation speed was increased every 30 sec by 4 rpm. Five mice were tested at the same time, separated by large disks. A trial starts when the mouse is placed on rotating rod, and it stops when the mouse falls down or when 5 min are completed. Falling down activates a switch that automatically stops a timer. The testing day, each mouse is submitted to 5 trials with an intertrial interval of 30 min. Mice housed five per cage were acclimated to a 12 h shift in light/dark cycle so that the exercise occurred during the animals normal wake period. Saline- and MPTP-treated mice fed with a control or HCT1026 diet (10/experimental group) were assessed for their Rotarod performance on day -7, +1, + 3 and +7 dpt.

### High-affinity [^3^H]dopamine uptake assay

Left and right striata were homogenized in ice-cold pre-lysis buffer (10 mM Tris, pH 7.5, and 0.32 M sucrose) using a Teflon pestle-glass mortar and homogenized tissue centrifuged for 10 min at 1000 × g at 4°C to remove nuclei. The supernatant containing the synaptosomes was collected and aliquots removed for the determination of protein content [71] and dopamine uptake (total high affinity and mazindol *non*-inhibitable). Fifty microliters of supernatant were diluted in Krebs-Ringer phopshate buffer (16 mM NaH_2_PO_4_,16 mM Na_2_HPO_4_, 119 mM NaCl, 4.7 mM KCl, 1.8 mM CaCl_2_, 1.2 mM MgSO_4_, 1.3 mM EDTA, and 5.6 mM glucose; pH 7.4), and incubated at 37°C in the presence or absence of mazindol (10 μM), a high affinity dopamine up-take inhibitor [8]. [^3^H]Dopamine (25 nM, specific activity, 20-40 Ci/mmol; Amersham, Arlington Heights, IL) was added in Krebs-Ringer buffer and incubation carried on for 6 min at 37°C. Synaptosomes were collected on presoaked nitrocellulose filters by filtration and *non*-specific radioactivity was washed with Krebs-Ringer phosphate buffer followed by filtration. The filters were then transferred into scintillation vials and measured by liquid scintillation (Cytoshint; ICN, Costa Mesa, CA) counter (Packard). Specific high-affinity neuronal dopamine uptake is expressed as fentomoles of dopamine uptake per microgram of protein minus the fentomoles of mazindol uptake. Values are represented as % changes in dopamine uptake vs. control.

### Measurement of MPP^+ ^levels in the striatum

Mice fed with the different diets were killed 90 min after MPTP injection. Both left and right striata were dissected on ice, placed into a vial containing 250 ul of 0.4 N perchloric acid and sonicated. After centrifugation, MPP^+ ^determinations were carried out by HPLC using 5SCX column (Phenomenex). The mobile phase was composed of H2S04 0.1 M, triethylamine 0.075 M, and acetonitrile 10% at pH 2.3; the flow was 1.5 ml mn^-1 ^[8].

### Immunohistochemistry

On the day of sacrifice, mice were anesthetized by intraperitoneal injection of Nembutal (50 mg/kg). Mice were rapidly perfused transcardially with 0.9% saline, followed by 4% paraformaldehyde in phosphate buffer (pH 7.2 at 4°C). Brains were carefully removed and post-fixed for 2-4 hrs, in 4% paraformaldehyde in phosphate buffer saline, pH 7.2 (PBS) and later placed in 15% sucrose in PBS overnight at 4°C. Tissues were frozen at -80° C. Serial cryostat sections (10 μm, from the olfactory bulb to the end of the medulla), were collected, mounted on poly-L-lysine-coated slides and processed for immunohistochemistry. Identification of the level was made by comparison with the sections of the mouse brain [72]. All immunostaining procedures were carried out on sections incubated in blocking buffer (0.3 - 0.5% Triton X-100, 5% BSA and 5% normal serum in PBS) for 30 min at room temp followed by an overnight incubation with the following primary antibodies in blocking buffer at 4°C: (i) tyrosine hydroxilase (TH) (goat anti-TH, Santa Cruz Biotechnology, Inc. USA 1:1000; rabbit anti-TH Pelfreez, Roger, AR, 1: 2000); (ii) rat anti dopamine transporter DAT, Millipore Corp. USA, 1:1000), as markers of dopaminergic neurons; (iii) membranolytic attack complex of complement (rat anti Mac-1/CD11b, Pharmingen International, Becton Dickinson, USA or anti Mac-1, Serotec, Oxford, UK, 1: 1000), as microglial marker; (iv) inducible nitric oxide synthase (rabbit anti-iNOS, Santa Cruz Biotechnology, Inc, 1: 200), 3-nitrotyrosine (rabbit anti-3-NT, Upstate, Lake Placid, NY, US, 1: 200), as NO/peroxynitrite finger-print. The sections were counterstained with nuclear counterstain (Dapi or PI, by Vector Laboratories Inc. Burlingame, CA, USA).

All antibodies, whether used for single or dual labeling procedures, were visualized by immunofluorescence, except for TH-Ab that was also visualized using immunoperoxidase. Adjacent tissue was also stained with cresyl violet to validate TH neuron survival [8,73,74]. Sections were incubated with the indicated dilutions of the antibodies, either alone or in combination as described. After 3 (× 5 min) washes in PBS, primary antibodies were revealed with specific FITC and CY3 conjugated secondary antibodies 1:100-1:200 dilution. (60 min at room temp). After 3 (× 5 min) washes in PBS, sections were mounted with Gel mounting solution (Biomeda corp. Foster City, CA, USA). In all of these protocols, blanks were processed as for experimental samples except that the primary antibodies were replaced with PBS.

### Loss of TH-positive neurons and striatal DAergic innervation

Loss of TH-positive (TH^+^) SNpc neurons was determined by serial section analysis of the total number of TH^+ ^cells counted throught the entire rostro-caudal (RC) axis of the murine SNpc (Bregma coordinates: -2.92, -3.08, -3.16, -3.20, -3.40 and -3.52) according to Franklin and Paxinos [72] at 7, 21, 30 and 40 days post-MPTP (dpt) or saline injection [8]. Cell counting was done in both side of the brain for each animal, and then right and left values were added to generate a total DA SNpc neuron count, in a total of five animals per experimental group. TH-labeled neurons were scored as positive only if their cell-body image included well defined nuclear counterstaining. Estimates of total TH^+^-stained and cresyl-violet-stained neurons in the SNpc were calculated using the Abercrombie's correction [74]. The total number of TH^+ ^cell bodies was estimated and examined by two independent researchers, in a blind fashion. Loss of striatal DAergic innervation was assessed by quantification of TH- and DAT-immunofluorescent (IF) signal intensity in 10 μm-thick coronal sections located at 0.5, 0.8 and 1.1 mm from bregma, and analyses carried out by confocal laser miscroscopy as described [8].

### Confocal laser microscopy and image analysis

Sections labeled by immunofluorescence were visualized and analyzed with a confocal laser scanning microscope LEICA TCS NT (Version 1.0, Leica Lasertechnik GmBH, Heidelberg, Germany), equipped with an argon/krypton laser using 10 ×, 20 ×, and 40 × and 100 × oil-immersion objectives. Pinhole was set at 1-1.3 for optical sections of 0.48-0.5 μm. For TH^+ ^and DAT^+ ^fibers in striatum, fluorescence intensity per unit of surface area was determined in 10 randomly selected fields (250.000 μm^2^) using computer-assisted image analysis software (LEICA). Single lower power scans were followed by 16 to 30 serial optical sectionings. Laser attenuation, pinhole diameter, photomultiplier sensitivity, and off-set were kept constants. The average fluorescence intensity (pixel, mean ± SEM per unit surface area) was measured throught the stack. Within the same stacks, the background pixel intensity in areas devoid of fibers/cells was determined and substracted. For assessment of reactive microglial cell number, ameboid-shaped Mac-1^+ ^cells [75] were counted in striatal and SNpc coronal sections, cell counts averaged for each animal and the mean number of cells per mm^2 ^per animal was estimated. A comparable countable area ranging from 1.90 mm^2 ^to 2.00 mm^3 ^was analyzed in the different MPTP groups. Double-labelled cells with iNOS and Mac-1, were counted and expressed as above. Dual stained TH^+ ^3-NT^+ ^cells were counted and values expressed as a percent double-stained TH^+ ^NT^+ ^/TH^+ ^neurons. Each label was analyzed on a total of 12 sections per mice and in at least 4 mice per group. Analyses were performed by two independent researchers blind to the experiment.

### Semi-quantitative RT-PCR

To analyze transcript levels, total RNA was isolated from striatum and ventral mid brain using the RNeasy isolation Midi kit (Qiagen, #75144). Tissue samples were homogenized in 1 ml of QIAzol Lysis Reagent (Qiagen, #79306) using a rotor-stator homogenizer. Total RNA was isolated from homogenized tissue samples using RNeasy Lipid Tissue Kit (Qiagen, #74804) including DNase digestion. At the end, RNA samples were redissolved in 30 μl of RNase-free water and their concentrations were determinated spectrophotometrically by A_260 _(Nanodrop-ND 1000). The cDNA was synthesized from 2 μg of total RNA using the Retroscript Kit (Ambion, #AM2224) following the manufacturer's directions. 250 ng of cDNA were used for PCR (96°C for 1 min for 2 cycles; 96°C for 1 min, 58°C for 4 min; 94°C for 1 min, 58°C for 2,5 min for 35 cycles, with a final extension at 70°C for 10 min) by using Super Taq DNA polymerase (Ambion, #AM1710) with specific primer pairs for TH (F: cgtggaatacacaaaggagg; R:ggtaggtttgatcttggtag; amplicon: 620 bp); DAT (F:cagagaggtggagctcatc; R:ggcagatcttccagacacc; amplicon: 328 bp), iNOS (F: tgctcccttccgaagtttctggcagcagcg; R: tcagagcctcgtggctttgggctcctc, amplicon: 500 bp) and Classic S18 Standard (amplicon: 495 bp; #Ambion AM1720), to normalize the expression. Samples from PCR reactions were separated electrophoretically on 2% agarose gel containing 0,2 μg ml^-1 ^of ethidium bromide. Fluorescent bands of amplified gene products were captured by using Gel Logic 200 Imaging System (Kodak), values normalized against S18, and ratios expressed as percent of control (saline-injected), within each experimental group.

### Western blot analysis

Protein extracts were prepared for striatum and ventral midbrain (which included the SNpc) (left and right sides) at the indicated time-intervals after saline or MPTP injections (n = 3 per group). The tissue samples were homogenized in lysis buffer (0.33 M sucrose, 8 mM Hepes, pH 7.4 and protease inhibitors) and quantified using the BCA protein determination method (Bio-Rad, Hercules, CA). Protein samples were diluted to equivalent volumes containing 20 μg of protein and boiled in an equal volume of Laemli SDS boiling buffer (Sigma) for 10 min. Samples were loaded into a 9-12% SDS-polyacrilamide gel and separated by electrophoresis for 3 h at 100 V. Proteins were transferred to polyvinylidene difluoride membrane (Amersham Biosciences, Piscataway, NJ) for 1.5 h at 300 mA. After blocking of nonspecific binding with 5% nonfat dry milk in TBST, the membranes were then probed with the following primary antibodies: rabbit anti-TH (Chemicon); rat anti-DAT (Millipore), rabbit anti-Mac1 (AbCam), mouse anti-gp91phox (BD Transduction Laboratories), β-actin (Cell Signaling). After incubation at room temperature for 1 hr, membranes were washed and treated with appropriate secondary antibodies conjugated with horseradish peroxidase (HRP) and blot were exposed onto radiographic film (Hyperfilm; Amersham Bioscience). Membranes were reprobed for β-actin immunolabeling as an internal control. The bands from the Western blots were densitometrically quantified on X-ray films (ImageQuantity One). The data from experimental band were normalized to β-actin, before statistical analysis of variance and values expressed as % of saline-injected controls.

### Statistical analysis

Data were analyzed by means of two-way analysis of variance (ANOVA), with group and time as independent variables and given as mean±SEM. Striatal neurochemical data (nmol or pmol mg protein^-1^) are expressed as % of controls. Comparisons *a posteriori *between different experiments were made by Student-Newman-Keuls t-test.

## Results

### HCT1026 preventive administration counteracts MPTP-induced down-regulation of high affinity synaptosomial DA uptake in the striatum

**Figure 1:**
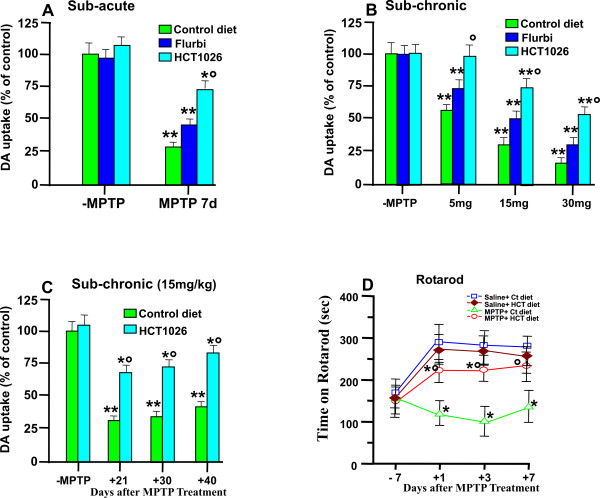
**HCT1026 inhibits MPTP-induced loss of high affinity synaptosomial [^3^H]DA uptake and reverses motor impairment**. Young and ageing C57Bl/6 mice fed with a control, flurbiprofen or HCT1026 diets (30 mg kg^-1^) starting at -10 d, underwent an MPTP treatment according to the subacute (**A) **or subchronic (**B)**, injection paradigms, as described. Age-matched mice fed with the different diets received physiologic saline (NaCl, 10 ml kg^-1^) and served as controls. Seven days after MPTP discontinuance, loss of DAergic functionality was assessed in striatum measuring high affinity synaptosomial striatal [^3^DA] uptake [8]. HCT1026 prooved to be more potent than its parent compound in counteracting MPTP-induced decreases in striatal DA uptake levels in both the subacute (**A**) and subchronic (**B**) protocols. Differences were analyzed by ANOVA followed by Newman-Keuls test, and considered significant when p < 0.05. **p < 0.05 vs saline, ° p < 0.05 vs MPTP + control diet. **C**. Ageing mice fed with a control or HCT1026 diets, were submitted to the subchronic MPTP regimen, and striatal DA uptake levels measured 21, 30 and 40 d after MPTP (n = 6/time point). Note the long-lasting counteraction of MPTP-induced striatal toxicity in mice fed with HCT1026 as opposed to the control diet. **D**: Motor performances on Rotarod of saline- and MPTP-treated mice (n = 10/group) fed with a control or HCT1026 diets. Time of permanence on revolving bars (ordinate) are plotted against pre- and post-treatment days (5 trials/day) during which experiments were performed. Mean and SEM values are reported. Establishment of a motor deficit measured 1-7 dpt, is counteracted by HCT1026. Differences were analyzed as above. ** p < 0.05 vs saline; ° p < 0.05 vs MPTP + control diet.

We first assessed the short-term effect of the control and medicated diets on striatal high affinity synaptosomial [^3^H]DA uptake (Figure 1A,B), a sensitive quantitative indicator of DAergic axonal terminal density [76]. In 2-3 month-old mice fed with flurbiprofen or HCT1026 diets and treated with saline, DA uptake levels were not different compared to saline-treated mice fed with the control diet. On the other hand, in mice fed with a control diet and exposed to the subacute MPTP (15 mg kg^-1^, 4 times a day, at 2 h intervals), we observed after 7 d the severe decrease (-75%) of striatal DA uptake (Figure 1A). In mice fed with flurbiprofen and treated with MPTP, a certain degree of protection was observed, as reflected by the less severe decrease (-54%) of DA uptake. On the other hand, HCT1026 afforded a greater protection, as illustrated by the significantly (p < 0.05) smaller (-25%) decrease of [^3^H] DA uptake levels as compared to mice fed with the control or flurbiprofen diets. With the subchronic (administration of MPTP at 24-h interval, for 5 consecutive days) regimen, increasing the daily doses of MPTP resulted in a dose-dependent loss of DA uptake levels (Figure 1B). By contrast, mice fed with HCT1026 and exposed to 5 mg kg^-1 ^day^-1 ^for five days, were resistant to MPTP-induced DAergic toxicity, as revealed by the counteraction of the almost 38-48% loss of striatal DA uptake measured in mice fed with the control or flurbiprofen diets. In addition, mice exposed to higher (15 and 30 mg kg^-1 ^day^-1^) MPTP doses and fed with control or flurbiprofen diets exhibited far greater (p < 0.05) decreases of striatal DA uptake compared with mice fed with HCT1026 (Figure 1B).

The preventive oral administration with HCT1026 was well tolerated and did not cause any measurable toxic effect throught the treatment, whereas flurbiprofen-fed mice showed severe gastrointestinal side-effects (bleeding). Due to flurbiprofen toxicity, only HCT1026 was studied in the long-term experimental protocol.

To more closely mimick PD condition, we next assessed the longterm efficacy and safety of HCT1026 in ageing (9-11 month-old) mice. Reportedly, the process of ageing increases DAergic vulnerability to MPTP and limits the repair capacity of the nigrostriatal DAergic system [77,78]. We thus selected the subchronic MPTP regimen, at a dose of 15 mg/kg [28,32,42]. Consistent with previous findings [77,78], ageing mice fed with a control diet did not recovered from MPTP insult, as revealed by an almost 70-75% decrease of DA uptake levels measured up to 40 dpt, whereas in mice fed with HCT1026, a significant degree of protection was measured, as reflected by the significant amelioration of striatal DA uptake levels at all time-points studied (Figure 1C). These data indicate that the neuroprotective activity of HCT1026 was maintained up to 40 dpt. In addition, the longterm administration of HCT1026 does not cause any measurable toxic effects.

### HCT1026 inhibits MPTP-induced motor impairment

To verify the ability of HCT1026 to affect MPTP-induced impairment of motor coordination [42,79,80], we assessed the ability to maintain the balance on a rotating cylinder and to adapt to the rate of locomotor activity, by using the Rotarod test, as described (Figure 1D). Saline- and MPTP-treated mice fed with a control or HCT1026 diet (n = 10/experimental group) were assessed for their Rotarod performance one week before saline or MPTP treatment (day -7) and + 1, + 3 and + 7 d post-MPTP. Because of the high degree of challenge of this task, mice of saline injected groups (-MPTP) fed with either a control or HCT1026 diet, performed better on the second trial (+ 1 d) and subsequent days, compared with d -7. By contrast, MPTP-treated mice fed with a control diet exhibited a significant decrease in the mean latency to fall at all time-points tested, compared to saline-injected mice (p < 0.05), defining a motor deficit in MPTP-treated animals (Figure 1D). In MPTP mice fed with HCT1026, the mean latency to fall was significantly (p < 0.05) increased compared to MPTP mice fed with a control diet at all time tested. By 7 dpt, HCT1026-fed mice performed as good as the control mice, indicating a significant reduction of the motor impairment by the preventive treatment with HCT1026.

### HCT1026 inhibits MPTP-induced loss of striatal TH and DAT at mRNA and protein levels

**Figure 2:**
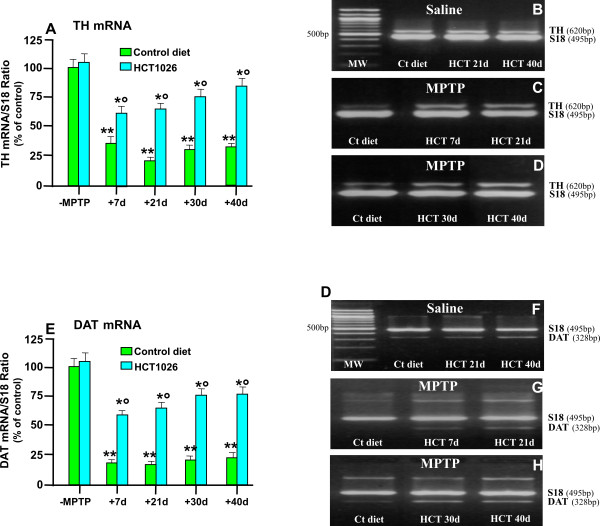
**HCT1026 inhibits MPTP-induced loss of striatal TH and DAT mRNAs expression**. Ageing (9-11 month-old) C57Bl/6 mice fed with a control (ct) or HCT1026 diets (30 mg kg^-1^) starting at -10 d, underwent an MPTP treatment according to the subchronic injection paradigm, as described. Age-matched mice fed with the different diets received physiologic saline and served as controls. Mice were sacrificed at different time-intervals after MPTP. Striatal tissue samples were processed for semi-quantitative RT-PCR analysis as described. Total RNA isolated and cDNA synthesized using Retroscript Kit (see Materials and Methods) following the manufacturer's directions. The 250 ng of cDNA were used for PCR, by using Super Taq DNA polymerase with specific primer pairs for TH (620 bp) and DAT (328 bp), and Classic S18 Standard (495 bp). Samples from PCR reactions were separated electrophoretically on 2% agarose gel containing 0,2 μg/ml of ethidium bromide (B-D, F-H). Fluorescent bands of amplified gene products were captured by using Gel Logic 200 Imaging System (Kodak), values normalized against S18 and ratios expressed as percent of control, within each experimental group (A, E). Differences were analyzed by ANOVA followed by Newman-Keuls test, and considered significant when p < 0.05. ** vs saline; ° p < 0.05 vs MPTP + control diet. Note the marked and long-lasting downregulation of TH (A,B,C,D) and DAT (E,F,G,H) mRNA transcript levels in striatal samples from ageing mice submitted to the subchronic MPTP regimen and the significant counteraction afforded by HCT1026.

Tyrosine hydroxylase (TH) is the rate-limiting enzyme in dopamine biosynthesis and a marker for DA neurons. The dopamine transporter, DAT, is a highly specific marker of projecting DAergic nigrostriatal neurons and thus, its expression is proportional to the loss of striatal dopamine content [76]. Accordingly, we examined TH and DAT striatal expression using RT-PCR, immunohistochemistry coupled to confocal microscopy, and western blot (WB) analyses. RT-PCR of TH (Figure 2A and 2B) and DAT (Figure 2E and 2F) mRNAs in striatum indicated no significant differences in transcript levels in saline-treated mice fed with control or HCT1026 diets. By contrast, exposure to MPTP induced a significant and longlasting decrease of TH mRNA in ageing mice fed with a control diet after either 7, 21 d (Figure 2A and 2C), 30 or 40 d (Figure 2A and 2D). In analogy with these findings, DAT mRNA levels were sharply down-regulated at 7, 21 (Figure 2E and 2G), 30 and 40 d (Figure 2E and 2H) post- MPTP in mice fed with a control diet. By contrast, in mice fed with HCT1026, TH and DAT mRNAs (Figure 2A, B, C and 2D) and DAT mRNA (Figure 2E, F, G and 2H) levels were significantly increased compared with those measured in MPTP mice fed with a control diet, thus supporting HCT1026-induced striatal DAergic neuroprotection was maintained at long time intervals.

**Figure 3:**
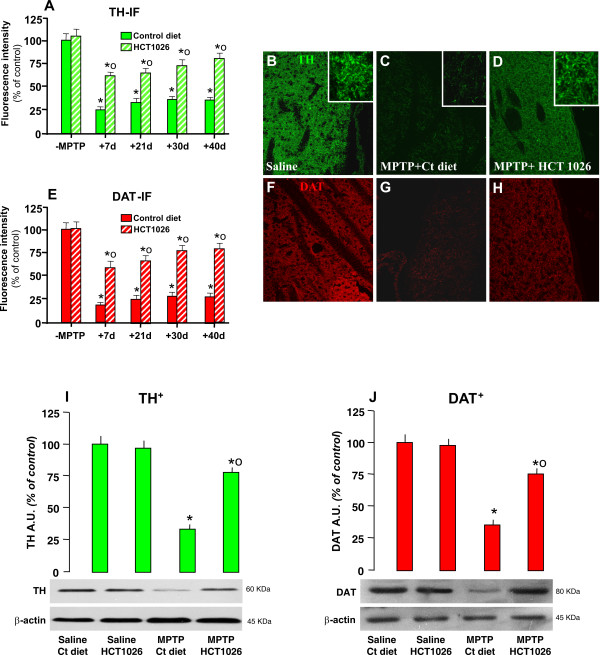
**HCT1026 inhibits MPTP-induced loss of striatal TH- and DAT- proteins by immunohistochemistry and western blotting**. **A**geing C57Bl/6 mice fed with a control (ct) or HCT1026 diets starting at -10 d, underwent an MPTP treatment, as described. At different time-intervals, mice were anesthetized and rapidly perfused, the brains were carefully removed and processed for immunohistochemistry, as described. TH- (A) and DAT-(E) IR in striatum (Str) assessed by immunofluorescent staining and image analysis by confocal Laser microscopy in ageing mice fed with ct or HCT1026 diets, treated with saline or MPTP (n = 5/time point). Fluorescence intensity values (FI, means ± S.E.M.) are expressed as % of saline. **p < 0.05 vs saline, °p < 0.05 vs MPTP fed with control diet. **B-H**: Representative confocal images show loss of TH-IF (revealed by FITC, green) in Str of MPTP mice fed with a ct diet at 40 dpt (C) and a substantial rescue of TH- (D) by HCT1026. **F-H**: Representative confocal images show loss of DAT-IF (revealed by FITC, green) in Str of MPTP mice fed with a ct diet at 40 dpt (G) and a substantial rescue of DAT-IF (H) by HCT1026. **E-F**: For western blot analysis, at 40 d after saline or MPTP injections in mice fed with the ct or HCT1026 diets, mice were sacrificed and striatal tissue samples processed for WB, as described. The data from experimental bands were normalized to β-actin, before statistical analysis of variance and values expressed as % of saline-injected controls, within each respective group. Note the significant decreased TH (I) and DAT (J) protein levels in MPTP mice fed with a ct diet, whereas a recovery was observed in HCT1026 fed mice. *p < 0.05 vs saline; *° p < 0.05 vs MPTP fed with ct.

As observed (Figure 3A and 3E), average striatal TH- and DAT-immunofluorescent (IF) signal intensity (pixel ± SEM), did not differ in saline-injected mice fed with either control or HCT1026-medicated diets. Subchronic MPTP treatment decreased TH- (Figure 3A and 3C) and DAT-IF (Figure 3E and 3G). In addition, loss of striatal DAergic innervation lasted up to 40 dpt. By contrast, HCT1026 significantly counteracted the loss of TH (Figure 3A and 3D)- and DAT (Figure 3E and 3H)-IF signal intensity, up to 40 dpt. Changes in TH and DAT proteins were next quantified by WB in saline and MPTP mice fed with the control or HCT1026 diet (Figure 3I and 3J). After 40 d from from MPTP administration, a significant reduction of MPTP-induced downregulation of both markers was observed only in mice fed with HCT1026 diet. These and previous findings support the long-term efficacy of HCT1026 in preserving TH and DAT expression and function in striatum of ageing mice exposed to subchronic MPTP regimen.

### MPTP metabolism is not affected by HCT1026 preventive treatment

One of the first limiting factors in MPTP toxicity is the conversion of MPTP into MPP^+ ^by means of the monoamine oxidase B (MAO-B) enzymatic activity. MPP+ is known to gain access into neurons via DAT, and through this mechanism it is accumulated into DAergic cells causing selective toxicity [76]. The striatal levels of MPP^+ ^were then measured 90 min after MPTP injection in mice fed with the different diets and exposed to MPTP. There was no significant difference in striatal MPP^+ ^levels measured 90 min after MPTP injection in mice fed with either the control (110 ± 7 ng mg^-1 ^protein) or HCT1026 (124 ± 12 ng mg^-1 ^protein) diets, thereby indicating that the greater protection afforded by HCT1026 might not be attributed to poor MPP^+ ^metabolism.

### HCT1026 preventive administration decreases MPTP-induced loss of TH^+ ^cell bodies in SNpc

**Figure 4:**
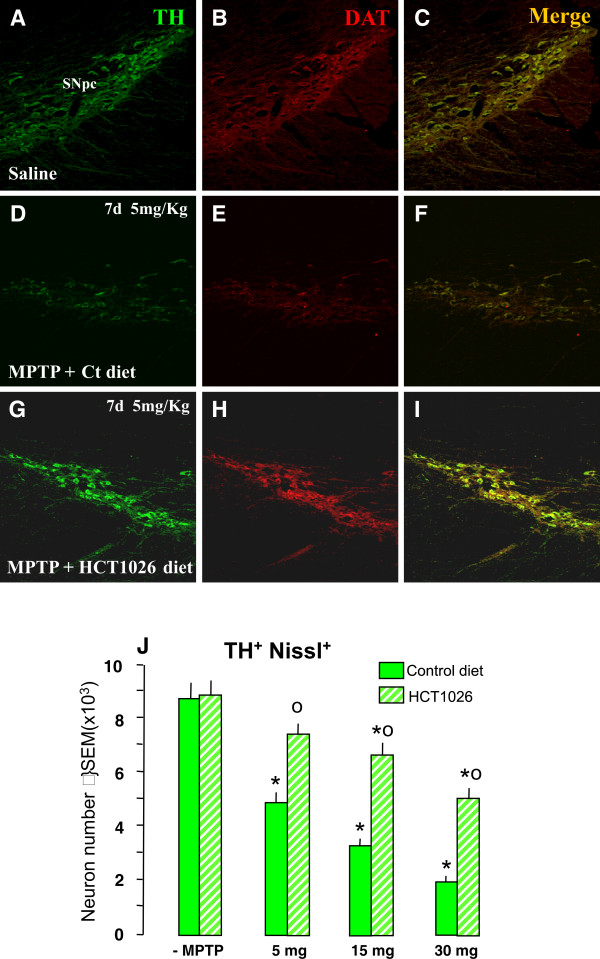
**HCT1026 preventive administration inhibits MPTP-induced dose-dependent loss of TH^+ ^cell bodies in SNpc**. Ageing mice fed with a ct or HCT1026 diets were submitted to the subchronic MPTP (5 mg, 15 mg or 30 mg kg^-1^, for 5 consecutive d) regimen, and DAergic cell survival studied after 7 d. **A-E**: Representative confocal images of dual staining with TH- (green) and DAT- (red) -Abs of coronal midbrain sections at the level of the SNpc 7 d after MPTP. Note the significant protection afforded by HCT10926 preventive treatment in mice treated with the 5 mg kg^-1 ^dose (see panels G-I) as compared to MPTP mice fed with a ct diet (D-F). **F**. Survival of DAergic cell bodies in SNpc. The total number of TH^+ ^and Nissl ^+ ^neurons was counted throught the entire rostro-caudal axis of the SNpc. Treatment groups were averaged (means ± S.E.M.) * p < 0.05 vs saline; *°p < 0.05, vs MPTP mice fed with a ct diet. HCT1026 significantly reduced the dose-dependent decrease of TH^+ ^and Nissl ^+ ^neurons.

**Figure 5:**
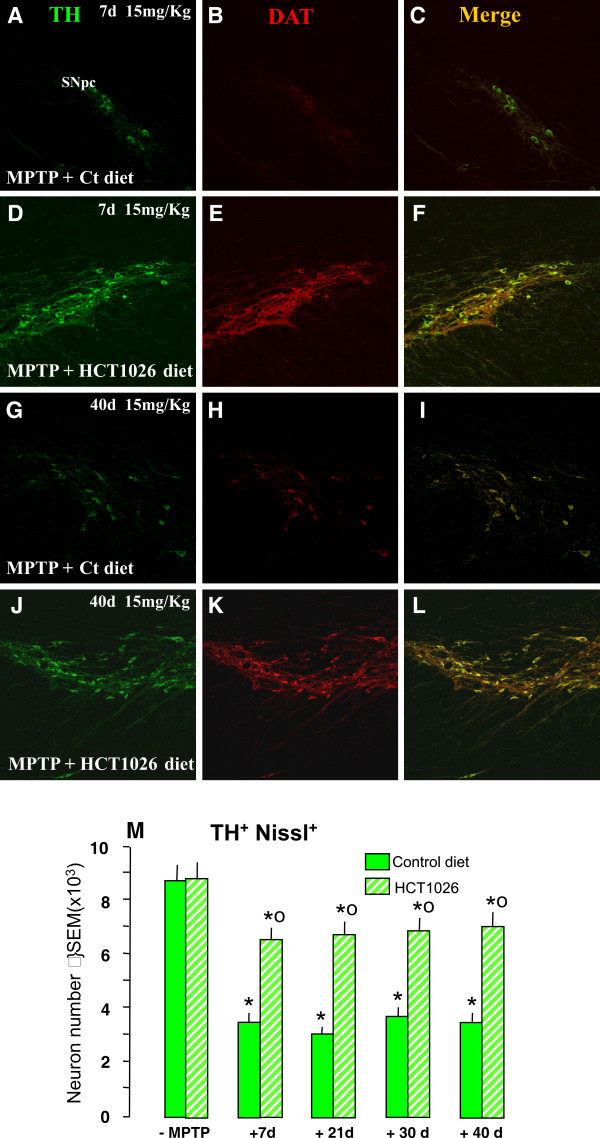
**Long-lasting protection of DAergic cell bodies in HCT1026 fed mice**. Ageing mice fed with a ct or HCT1026 diet were submitted to MPTP subchronic regimen (15 mg kg^-1 ^for 5 cosecutive d) and sacrificed at differet time-intervals after MPTP. **A-L**: Representative confocal images of dual staining with TH- (green) and DAT- (red) -Abs of coronal midbrain sections at the level of the SNpc 7 and 40 d after MPTP. As observed, MPTP mice fed with a ct diet show a marked loss of TH^+^DAT^+ ^neurons at 7 d (see panels A-C) and fail to recover 40 d following MPTP injury (G-I). By contrast, HCT1026 afforded a significant and lonlasting protection (see panels D-F and J-L). **M**: The total number of TH^+ ^and Nissl ^+ ^neurons was counted throught the entire rostro-caudal axis of the SNpc. Treatment groups were averaged (means ± S.E.M.) * p < 0.05 vs saline; *°p < 0.05, vs MPTP mice fed with a ct diet. HCT1026 significantly reduced the decrease of TH^+ ^and Nissl ^+ ^neurons observed up to 40 d in mice fed with a ct diet.

We next assessed the impact of HCT1026 in MPTP-induced toxicity of nigral DAergic cell bodies (Figure 4 and Figure 5). In mice fed with a control diet, MPTP induced a dose-dependent loss of double-stained TH^+ ^(revealed by FITC, in green) DAT^+ ^(revealed by CY3, in red) cells in SNpc (compare Figure 4A, B and 4C with Figure 4D, E and 4F), whereas MPTP-induced DAergic neurotoxicity was signicantly (p < 0.05) reduced in HCT1026-fed mice (Figure 4G, H and 4I). Estimation of the total number of TH^+ ^Nissl^+ ^neurons using the Abercrombie correction, confirmed a dose-dependent reduction of TH^+ ^Nissl^+ ^neurons in MPTP-treated mice fed with control diet, suggesting an actual TH^+ ^neuronal loss rather than loss of TH expression (Figure 4J). By contrast, HCT1026-fed mice exhibited a significantly greater number of TH^+ ^Nissl^+ ^neurons at all doses tested (Figure 4J). These results suggest that a certain number of DAergic neurons could survive the MPTP insult in mice receiving a preventive treatment with HCT1026.

In order to verify the ability of the continous oral treatment with HCT1026 to maintain its neuroprotective effects, saline and MPTP (15 mg kg^-1 ^day-^1 ^per 5 consecutive days), mice fed with the control or HCT1026 diet were sacrificed at 21, 30 and 40 dpt. As observed, at either 7 d (Figure 5A, B, C and 5M), 21, 30 (Figure 5M), or 40 dpt (Figure 5G, H, I and 5M) MPTP mice fed with the control diet did not recover as reflected by the significant, long-lasting loss of TH^+ ^Nissl^+ ^neurons. In sharp contrast, HCT1026 exerted a significant neuroprotective effect that was maintained up to 40 dpt (see Figure 5D, E, F, and 5J, K, L, M), suggesting increased survival/rescue of SNpc neurons in HCT1026-fed mice.

### HCT1026 inhibits MPTP-induced microglial activation

Glial inflammatory mechanisms are thought to contribute to MPTP-induced nigrostriatal DAergic degeneration (see Refs in Background). Indeed, when microglia adopts a pro-inflammatory phenotype, the production and release of a plethora of toxic mediators, including pro-inflammatory cytokines and iNOS-derived NO, can enhance neuronal damage in the SNpc and accelerate the appearance of behavioral symptoms [32,81-83]. The ability of HCT1026 to modulate MPTP-induced microglial activation was next assessed during the early phase of active degeneration [8] using immunohistochemistry, WB and RT-PCR analysis.

### Microglial cell number/morphology in striatum and SNpc

**Figure 6:**
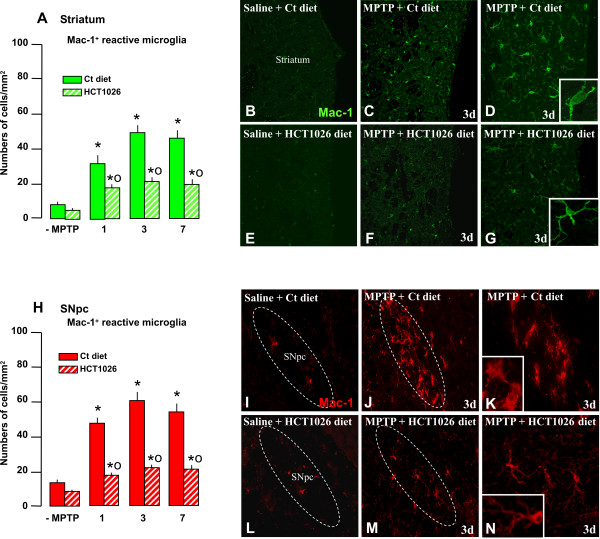
**HCT1026 inhibits MPTP-induced increased reactive Mac-1^+ ^microglia in striatum and SNpc**. Ageing (9-11 month-old) C57Bl/6 mice fed with a control (ct) or HCT1026 diets (30 mg kg^-1^) starting at -10 d, underwent an MPTP treatment, as described. At different time-intervals mice were anesthetized and rapidly perfused, the brains processed for immunohistochemistry. Coronal sections at the level of the striatum and SNpc were stained with Mac-1-Ab to localize microglial cells. A-B: Reactive (ameboid-like) microglial cells were counted at different time-intervals after saline and MPTP injection (n = 4/experimental group) in mice fed with a ct or HCT1026 diets, within both Str (A-G) and SNpc (H-N). Differences were analyzed by ANOVA followed by Newman-Keuls test, and considered significant when p < 0.05. **p < 0.05 vs saline; *° p < 0.05 vs MPTP mice fed with ct diet. **B-E**: Representative confocal images of Mac-1 staining (green) in saline (B), 3 d after MPTP in mice fed with ct diet (C, 20× and D, 40×) or HCT1026 (F, 20× and G, 40×). Insets (100×) show microglia morphologic appearance. **I-N**: Representative confocal images of Mac-1 staining (red) in SNpc. Note the high density of reactive Mac-1^+ ^cells with rounded cell bodies and short, thick processes 3 d after MPTP administration in mice fed with a ct diet (J-K), as compared to Mac-1 microglia of mice fed with HCT1026 (M-N), exhibiting a more elongated cell body and long ramified processes.

Changes in activated microglial cell number and morphologic appearance at both striatal and SNpc levels were assessed using Mac-1-Ab, an integrin receptor known to mediate reactive microgliosis and recognized to significantly contribute to the progressive dopaminergic neurodegeneration in the MPTP model of DAergic toxicity, both *in vivo *and *in vitro *[see [28-33] and background]. In saline-injected mice fed with either control or HCT1026 diets, Mac-1^+^-microglial cells with elongated cell bodies and ramified processes were present. As previously shown, an increased number of Mac1^+ ^cells with morphological characteristics of activated microglia (i.e. ameboid, round shaped Mac1^+ ^cells with thick and short processes) was observed in both striatum (Figure 6A, C, D and inset) and SNpc (Figure 6H, J, K and inset) levels. By contrast, Mac-1^+ ^cells exhibited a more elongated cell body with longer and thinner processes in mice fed with HCT1026 diet (see insets in Figure 6G and 6N). Moreover, the number of ameboid-like Mac-1^+ ^cells was sharply reduced both in striatum (Figure 6A, F and 6G) and midbrain (Figure 6H, M and 6N) of HCT1026-fed mice.

### Mac-1 and PHOX expression in the ventral midbrain (VM)

**Figure 7:**
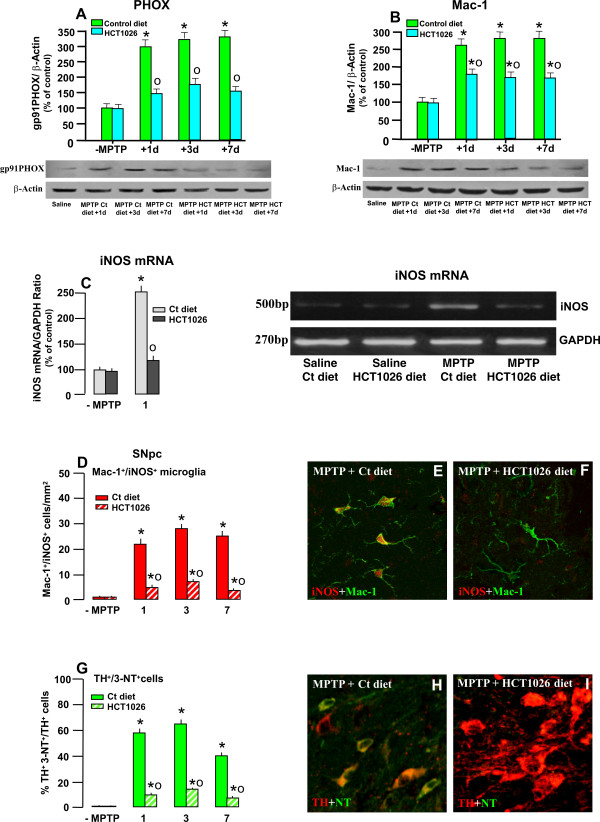
**HCT1026 inhibits MPTP-induced increased Mac-1, PHOX, iNOX and 3-Nitrotyrosine expression in SNpc**. **A-B: **Western blotting of phagocyte oxidase PHOX (A) and Mac-1 (B) within the VM at different time-intervals after saline or MPTP injection in mice fed with the control or HCT1026 diet. The data from experimental bands were normalized to β-actin, before statistical analysis of variance. Values are expressed as % of saline-injected controls. Differences were analyzed by ANOVA followed by Newman-Keuls test, and considered significant when p < 0.05. * p < 0.05 compared to saline; ° p < 0.05 vs MPTP fed with the control diet. **C**. Semi-quantitative RT-PCR for iNOS. The 250 ng of cDNA were used for PCR, by using Super Taq DNA polymerase with specific primer pairs for iNOS (500 bp) and Classic GADPH Standard (270 bp). Samples from PCR reactions were processed as described. Fluorescent bands of amplified gene products were analyzed, the values normalized against GADPH and ratios expressed as % of control, within each experimental group (C), see text for details. Differences were analyzed by ANOVA as above. ** p < 0.05 vs saline; ° p < 0.05 vs MPTP + control diet. **D**: Mean numbers of Mac1^+^iNOS^+ ^cells within the SNpc in saline and MPTP mice fed with the control or HCT1026 diet. Cell counts obtained as described in Material section. **E-F**: Representative confocal images showing double staining with Mac-1 (green) and iNOS (red) in MPTP mice fed with the control (E) or HCT1026 (F) diets. **G**: Percent (%) of TH^+ ^neurons colocalizing with 3-nitrotyrosine (3-NT), a peroxynitrite footprint. Dual stained TH^+ ^3-NT^+ ^neurons were counted as described and values expressed as % of total TH^+ ^neurons. **H-I**: Representative confocal images showing dual immunostaining with 3-NT (green) and TH (red) in MPTP mice fed with a ct diet (H) showing that a large proportion of DAergic neurons colocalize (orange-to-yellow) as opposed to TH neurons of mice fed with HCT1026 (E) where no colocalization was observed in the large part of SNpc neurons.

Mac-1 is linked to the activation of PHOX, a chief component of MPTP-dependent microglia activation [29-33]. Given that Mac-1 and PHOX act in a synergistic way, and are essential to enhance DAergic degeneration, we next evaluated the expression of both markers within the ventral midbrain by WB, during the maximal phase of glia activation [8]. As previously observed, a sharp increase of Mac-1 and PHOX followed MPTP treatment, as early as 1 dpt, in mice fed with the control diet, with levels remaining high at both 3 and 7 dpt (Figure 7A, B). Conversely, HCT1026 significantly reduced MPTP-induced Mac-1 and PHOX expression in the ventral midbrain, supporting reduced microglial activation during the early phase of DAergic degeneration (Figure 7A, B).

### Expression of pro-inflammatory mediators: iNOS and 3-NT

The ability of a preventive treatment with HCT1026 to inhibit MPTP-induced up-regulation of iNOS was next evaluated by semi-quantitative RT-PCR analysis 1 d after MPTP challenge. Within the ventral midbrain of saline-treated mice fed with a control or HCT1026 diet, no significant difference was observed in iNOS mRNA levels, whereas MPTP significantly up-regulated iNOS transcription (Figure 7C). On the other hand, the MPTP mice fed with HCT1026 exhibited significantly less iNOS mRNA expression compared to controls (Figure 7C).

When glial superoxide generated by PHOX activation and iNOS/NO are concomitantly active, peroxynitrite (ONOO-), and consequently protein tyrosine nitration and hydroxyl radicals over-production occurs [33]. Hence, the generation of high NO concentration followed by the production of peroxynitrite may be involved in DAergic neuronal cell death. We thus used immunohistochemistry to localize iNOS and 3-nitrotyrosine (3-NT) as fingerprint of iNOS-derived NO and peroxynitrite generation. Dual immunostaining with iNOS and Mac-1 within the SNpc of MPTP mice fed with a control diet, indicated that a significant proportion of reactive Mac-1 cells expressed iNOS, 1-7 d post-MPTP (Figure 7D, E). Dual staining with 3-NT- and TH indicated a sharp increase in the percentage of TH^+ ^cells colocalizing with 3-NT at both 1 and 3 d post-MPTP (Figure 7G, H), while a certain decrease was observed 7 dpt. Conversely, in MPTP mice fed with HCT1026, both iNOS- (Figure 7D, F) and 3-NT-(Figure 7G, I) were significantly downregulated 1-7 dpt within the SNpc.

These results indicated an efficient reduction of MPTP-induced microglial activation, pro-inflammatory marker expression, i.e. Mac-1, PHOX, iNOS and 3-NT formation within the SNpc of mice fed with HCT1026, which might account for the observed nigrostriatal protection.

## Discussion

Grafting a NO-donating moiety into the structure of flurbiprofen, one of the most potent non-selective anti-inflammatory agents, yielded a drug devoided of side toxicity and endowed with a remarkable neuroprotective activity against MPTP-induced DAergic neurotoxicity, motor impairment and microglia activation in ageing mice. HCT1026 was effective in both the acute and subchronic models of MPTP, and its neuroprotective activity lasted up to 40 d, as opposed to age-matched controls indicating the longterm safety and efficacy of the HCT1026, as opposed to flurbiprofen. The different outcome of mice treated with HCT1026 was not due to differences in daily food consumption, or to poor MPP^+ ^metabolism. HCT1026-induced neuroprotection was associated with a marked down-regulation of activated microglial cell number and a signiof ficant decrease of MPTP-induced pro-inflammatory mediators, including iNOS, Mac-1 and PHOX expression, as well reduced 3-NT formationin SNpc DAergic neurons, suggesting that a switch in microglia pro-inflammatory phenotype might contribute to nigrostriatal neuroprotection.

The experimental study was designed to compare the oral activity of HCT1026 with that of flurbiprofen. However, due to the significant gastric toxicity of flurbiprofen observed in the short-term study, only HCT1026-medicated diet was further studied. In long-term experimental protocols, HCT1026 proved to have a safe profile and a significant efficacy in counteracting MPTP-induced striatal DAergic toxicity.

Nitric oxide (•NO) is a biological molecule known to play a major role in a wide variety of physiological and pathological conditions [84]. Some of its functions include vasodilation of blood vessels, GI mucosal healing and defense. Therefore NSAIDs containing NO-donor groups have been developed to obtain effective treatment of inflammation with reduced GI side effects [57-61]. Indeed, grafting an organic nitrate moiety onto the NSAID scaffold has been shown to result in the release of NO through slow kinetics (in comparison with others NO donors, i.e. sodium nitroprusside, S-nitroso-N-acetyl-D,L, penicillamine), possibly mimicking the physiological levels of NO produced by constitutive NO synthases. Thanks to NO release, and the combination of a balanced inhibition of the two main COX isoforms, NO-NSAIDs are endowed with little gastrointestinal and renal toxicity compared to their parents compounds [56-61,65,85]. It is believed that the NO, which is released by the metabolism of nitrate as the compounds are broken down, may counteract the consequences of the NSAID-induced decrease in gastric mucosal PGs [65]. It seems important to mention that a clear identification of the metabolic steps by which NO-NSAIDs produce NO has not been established. Experimental findings obtained, *in vivo*, show that HCT1026 is metabolized into flurbiprofen and NO species, i.e. nitrates and nitrites, [60,61,70], which are detected in plasma and in brain at 2-4 h [70] and 3 h [86], respectively from drug administration. In vitro, HCT1026 is converted into flurbiprofen with different kinetics depending on the cell assay. In rat plasma, 30 min of incubation is required to fully convert HCT1026 to flurbiprofen [see [63]]. By contrast, approximately 35% of HCT1026 is converted into flurbiprofen within 1 h of incubation in human blood, and 24 h of incubation was necessary to reach the almost complete dissociation of HCT1026 [62,63]. Given the rapid action of HCT1026 demonstrated *in vitro*, Bernardo et al. [63] suggested the possibility that HCT1026 might reach the brain parenchyma and act on brain cells before being cleaved to the nitrate moiety and flurbiprofen [63]. Alternatively, the metabolites flurbiprofen and NO might act concomitantly by activating parallel pathways that ultimately determine the unique effects of HCT1026 [63]. Other studies have suggested a potential action of the HCT-1026 metabolite, 4-hydroxybutyl nitrate, since animal studies have shown that the level of inorganic nitrite in the brain increases after oral administration of HCT-1026 [86]. Plasma nitrite itself has been shown to provide a source of NO under certain conditions [see [87]].

Neurochemical, morphological and behavioral changes clearly indicate that with the ageing process, increased vulnerability of the nigrostriatal DAergic system and limited recovery from MPTP injury are observed [4,5,17,18,77,78]. Indeed, the nigrostriatal DAergic neurons exhibit compensatory mechanisms in response to MPTP injury, but the degree of plasticity becomes reduced with age [18,77,78]. Accordingly, a diminished compensatory capacity of nigrostriatal DAergic neurons "as a prelude" to PD is recognized to accompain the process of aging [see [88,89]]. Among the mechanisms at play, increased neuronal vulnerability to degenerative conditions, dysfunction of glia-neuron crosstalk, reduced repair capacity of injured DAergic neurons and/or limited neurogenesis may contribute to the poor recovery observed with age [see [18] for review]. Given the role of the ageing process as a critical risk factor for developing PD, we addressed the efficacy of HCT1026 preventive administration schedule in 9 to 11 month-old mice and found that longterm administration of HCT1026 resulted in a significant DAergic neuroprotection following MPTP insult, at both and SNpc levels. These effects lasted up to 40 dpt, supporting HCT1026 as promising approach towards the development of effective pharmacological neuroprotective strategies against PD.

Variable neuroprotective effects have been, so far, reported for both steroidal and non-sterodal, mixed and COX-2-selective inhibitors in different MPTP-mouse models of PD [37-48]. The mixed COX-1/COX-2 inhibitor indomethacine [39] and a COX-2 selective NSAID, rofecoxib [38] treatment rescued DAergic neurons from MPTP injury. Indomethacine, however, appeared toxic at high doses, and rofecoxib, failed to keep its protective properties when used in the prolonged treatment [90]. Studies on mice deficient with COX-2 showed that COX-2 plays a critical role in animal models of DAergic degeneration [38,32,43]. In particular, the role of increased levels of COX-2 in generating a toxic dopamine-quinone species which was responsible for DAergic neuronal degeneration, was demonstrated, whereas the selective COX-2 inhibitor, rofecoxib, exhibited a neuroprotective effect (38). In the study of Sanchez-Pernaute [43], the COX-2 antagonist, celecoxib, was capable to prevent or slow down DAergic degeneration induced by intrastriatal administration of 6-OHDA. Accordingly, Vijitruth et al. [42] showed that pharmacological or genetic inhibition of COX-2 was capable to reduce motor impairment and to protect DAergic neuronal cell bodies in the SNpc as well as the striatal TH-stained fibres against MPTP-induced neurotoxicity [42].

On the other hand, Ibuprofen, a non selective blocker, was shown to diminish the decline of dopamine content in striatum in the MPTP mouse model of PD, in a dose-dependent manner, and was not toxic to the DAergic system [41]. In accord with these experimental results, ibuprofen, but not other non-selective NSAIDs, was shown to diminish the risk/incidence of PD in men [5,6]. The present results showing the longterm DAergic neuroprotection in ageing mice and the safety profile of HCT1026 are of special interest, given that *non*-selective NSAIDs long-term therapies are hampered by their significant gastrointestinal, renal and cardiovascular side-effects [56,85].

HCT1026-induced neuroprotection observed in the present study was accompanied by a sharp downregulation of all studied markers of microglial activation including ameboid-like microglial cell number, pro-inflammatory mediators as well as two key harmfull elements, MAC-1 and PHOX, likely suggesting that a shift from microglial pro-inflammatory ("harmfull") phenotype might be a major contributing factor.

Indeed, under inflammatory conditions, PHOX is the major source of peroxides in the brain. Activation of microglial PHOX is synergistic with glial iNOS expression in inducing DAergic neuron death [27-36]. Accordingly, PHOX/Mac-1-deficiency mitigates MPTP-induced DAergic neurotoxicity both *in vivo *and *in vitro *[30-33]. Inflammatory stimuli together with ROS and RNS activate nuclear factor-kB (NF-kB) in microglial cells, oligodendrocytes and neurons to promote the transcription of inflammatory cytokines, COX-2, iNOS, and apoptosis-promoting factor including p53/Bax [see [45,46]]. Various studies (reported in Backgroud section) have clearly underlined a "dual key mechanism" whereby simultaneous activation glial iNOS and PHOX synergistically act in killing DAergic neurons [33]. This mechanism may mediate inflammatory degeneration in response to cytokines, bacteria, ATP, arachidonate, whereas neuroprotection was observed by NO and peroxynitrite scavengers [33]. Indeed, nitrative stress is among the factors potentially underlying DAergic neurodegeneration [8,27,28,33-36]. Interestingly, the number of DAergic neurons containing 3-NT increased significantly in rhesus monkey midbrain DAergic neurons with age, suggesting a role for aging-related increase of nitrative damage in the selective vulnerability of SN neurons to degeneration in PD [91]. Here, ageing mice fed with a control diet exhibited a dramatic increase of 3-NT colocalization with TH^+ ^neurons by 3 d after MPTP, corresponding to the active DAergic degeneration phase [27,28,32,33,36], while by 7 d a certain decrease in 3-NT accumulation within TH^+ ^neurons was observed, possibly indicating the end of the degeneration phase. In HCT1026-fed mice, the generation of PHOX and iNOS-derived cytotoxic mediators, including 3-NT accumulation within DAergic neurons, were markedly abated, supporting that a significant proportion of SNpc neurons survived MPTP insult.

Although NSAIDs pharmacological actions are related to their ability to inhibit PG biosynthesis, some of their beneficial therapeutical effects are thought to be mediated by a panel of COX-independent mechanisms. NSAIDs are able to inactivate the transcription NF-kB and activator protein-1 (AP-1), critically involved in the induction of multiple inflammatory gene products involved in the inflammatory response (i.e. iNOS, TNF-α). In addition, NSAIDs in neuronal cells might directly and dose-dependently scavenge ROS and RNS, thereby blocking their detrimental effects [45,46]. On the other hand, high concentrations of NSAIDs such as ibuprofen and indomethacin, activate PPARγ. PPARγ is a ligand activated inhibitory transcription factor that antagonizes the activity of NFkB, AP1, signal transducer and activator of transcription-1 (STAT-1) and nuclear factor of activated T cells (NFAT). PPARγ activation is then associated with a reduction in the expression of several inflammatory genes and the production of inflammatory cytokines and iNOS [45,46]. In particular, *in vitro *studies have reported that selective PPARγ agonists such as pioglitazone, ibuprofen, or indomethacin, can activate PPARγ in microglia, decreasing the number of activated glial cells [see [45,46]].

The mechanisms that differentiate HCT-1026 from flurbiprofen remains a matter of debate. *In vitro *studies demonstrated that a low concentration of (1 μM) of HCT1026, but not flurbiprofen, activated PPARγ in primary cultures of rat microglia, with kinetics similar to those of the synthetic agonist, ciglitazone [63], supporting additional anti-inflammatory action through PPARγ [63]. In addition PPARγ agonists were reported to mitigate MPTP-induced DAergic neurotoxicity in different PD models [see [13-15,45,46]]. In the recent studies of Abdul-Hay et al. [87], flurbiprofen was 10-fold less potent than HCT-1026 in inhibiting iNOS induction in RAW 264.7 cell cultures. In LPS/IFNγ-induced primary astroglial cultures, HCT-1026 showed anti-inflammatory potency towards inhibition of cytokine and iNOS elevation, providing similar observations to those in microglial cultures [62]. That the anti-inflammatory activity of HCT-1026 could translate into neuroprotection was further demonstrated in a co-culture experiment with LPS-stimulated RAW cells and a neuroblastoma cell culture, where HCT-1026 was highly efficacious neuroprotectant [87].

Grafting a NO-donating moiety to flurbiprofen was reported to confer additional anti-inflammatory properties [51,52,60-66,87,92,93]. It has been suggested that this effect may depend on the negative feedback regulation exerted by low physiologic concentration of NO (nanomolar range) on different inflammatory mediators such as iNOS and COX-2, as well as on their associated functions [94-97]. Indeed, there is evidence that at low concentrations, NO has anti-inflammatory properties as it inhibits the expression of pro-inflammatory proteins (i.e. COX-2 and iNOS), and it counteracts the release of pro-inflammatory cytokines, such as TNF-α, in activated macrophages [94-97].

It should be recalled, that NO signaling plays an important role in the functioning of the CNS, and activation of soluble guanylate cuclase (sGC) represent one important effect of NO. Of note, physiological release of low concentrations of NO by constitutive neuronal NOS is recognized to modulate extracellular levels of dopamine in the striatum and to critically participate in striatal DAergic homeostasis [98]. The NO/sGC/cGMP signal transduction system is also considered to be important for modulating synaptic transmission and plasticity in brain regions such as the hippocampus, cerebral cortex, and cerebellum, and further studies are required to unravel potential involvement of these pathways in DAergic neuroprotection afforded by HCT1026. Besides the NO-mediated effects, most recently proposed are NO-independent and NSAID-independent actions on NFκB and MAPK/ERK signaling pathways [see [64]]. In their study, Idris and co [64] reported the ability of HCT1026 to inhibit receptor activator of NFkB (RANKL), as well as RANKL-induced activation of NFkB and ERK pathway in LPS-stimulated macrophage cultures. In addition, HCT1026 also inhibited TNF-α, IL-1 and LPS-induced signaling. Interestingly enough, the pathways inhibited by HCT1026 all share a similar kinase complex upstream of the NFkB and ERK pathways and this is the most likely target for the action of HCT1026 [64].

It seems important to underline that inflammatory pathways may become hyperactivated with age and/or become more sensitive to immune/neurotoxic challenge, thereby promoting degeneration [99-102]. Given that with age, dysfunctional microglia and altered glia-neuron crosstalk may contribute to the progression of neuronal degeneration [18], HCT1026 preventive and long-term treatment might thus reduce age-dependent and MPTP-induced increase in oxidative and inflammatory attacks to nigrostriatal DAergic neurons. Of special interest, in view of the role of both systemic and central inflammation in modulating the severity of neuronal insult, including DAergic injury [see [13-15,18,24,81-83]], a potential effect of HCT1026 in influencing systemic inflammation cannot be excluded. In addition, the mitigation of the nitrosative/oxidative status of the nigral microenvironment as revealed by downregulation of Mac-1, PHOX and 3-NT in the VM, likely have beneficial consequences for glial expression of critical neuroprotective/neurotrophic factors [18], thereby supporting TH^+ ^neuron survival/neurorescue, possibly through an amelioration/mitigation of SNpc nicroenvironment.

After brain injury, the inflammatory environment is recognized to have both detrimental and beneficial effects on neuronal outcome, depending on mouse strain, age and sex of the host, the severity of the lesion, the degree and timing glial activation, the hormonal background, the specific cellular context and intrinsic region-specific neuronal characteristics [see [8-18,21-26,40,77,78,100,103,104]]. In degenerative conditions, glia serve neuroprotective functions including the removal of dead cells by phagocytic activity and the production of neurotrophic factors. By contrast, overactivation of microglia or dysfunctional microglial cells as a consequence of ageing and age-related events within the SN microenviroment, [18,25,100-102] likely increase DAergic neuron vulnerability and/or may limit DAergic self-repair abilities. *In vivo *experiments have recently shown that intranigral administration of prostaglandin J2 (PJ2) induces microglia activation, selective degeneration of DAergic neurons in the SNpc, formation of ubiquitin- and α-synuclein-immunoreactive aggregates in the spared DAergic neurons, and locomotor deficit [83]. These and other findings have underlined the role of a transient initiation factor, triggering an active self-perpetuating cycle of chronic neuroinflammation, contributing to DAergic neuronal dysfunction [13,83]. By reducing exacerbation of inflammation, HCT1026 may then improve mitochondrial performance, increase glial-mediated neurotrophic support, thus creating a more favorable milieu for nigrostriatal DAergic neuron survival/rescue.

The present results are in line with data obtained in different animal models of brain inflammation, where HCT1026 significantly reduced neuronal loss and decreased the number of reactive microglial cells to a greater extent than the parent compound, flurbiprofen [66,86,87,92,93]. In experimental allergic encephalomyelitis (EAE), oral treatment with HCT1026 which delayed disease onset and decreased the severity of clinical signs in mice immunized with myelin oligodendrocyte peptide (MOG35-55) [66]. In addition, HCT1026 fed mice exhibited significantly reduced mRNA levels of pro-inflammatory cytokines, caspase-1, and iNOS in blood cells, with reduced number of CNS-infiltrating T cells [66]. Recently, HCT1026 was reported to mitigate amyloid-β-induced toxicity, in cell culture, *in vitro*, while enhancing cognition in response to cholinergic blockade, *in vivo *[87]. Other studies reported the ability of HCT1026 to reduce microglia activation and to prevent muscular dystrophy pathology in two murine models [93].

## Conclusions

We herein report that oral preventive administration of the NO-donating derivative of flurbiprofen, HCT1026, has a safe profile and a significant efficacy in counteracting MPTP-induced DAergic neurotoxicity, motor impairment and microglia activation in ageing mice. In particular, nigrostrial DAergic neuroprotection afforded by HCT1026 lasted for 40 d after MPTP administration. Hence, DAergic neurons exhibited an increased ability to resist to the cytotoxic environment caused by MPTP injury, leading to a significant neurorescue observed within the striatum and SNpc, at a morphological, neurochemical, and molecular levels. These effects of HCT1026 were associated with reduced microglial proinflammatory phenotype and reduced formation of the peroxynitrite footprint, 3-NT, within TH^+ ^cell bodies. While further studies are required to clarify the mechanism(s) of HCT1026 neuroprotective effects, the combination of a balanced inhibition of the two main COX isoforms with NO release provides a promising approach towards the development of novel and effective therapeutic strategies against PD.

## List of abbreviations

(3-NT): 3-nitrotyrosine; (COX-2): Cyclooxygenase-2; (DA): Dopamine; (DAT): Dopamine transporter; (EAE): Experimental Allergic Encephalomyelitis; (GR): Glucocorticoid receptor; (HCT1026): [2-fluoro-α-methyl(1,1'-biphenyl)-4-acetic-4-(nitrooxy)butyl ester]; (HRP): Horseradish peroxidase; (IF): Immunofluorescence; (iNOS): Inducible-nitric oxide synthase; (LPS): Lipopolysaccharide; (Mac-1): Macrophage antigen-1; (MAO-B): Monoamine oxidase B; (MOG35-55): Myelin oligodendrocyte peptide; (MPP^+^): 1-methyl-4-phenylpyrdinium ion; (MPTP): 1-methyl-4-phenyl-1,2,3,6-tetrahydropyridine; (NO): Nitric oxide; (NSAIDs): Nonsteroidal anti-inflammatory drugs; (ONOO^-^): Peroxynitrite; (PD): Parkinson's disease; (PGs): Prostaglandins; (PHOX): NADPH oxidase; (PJ2): Prostaglandin J2; (PPAR-γ): Peroxisome proliferator-activated receptor-γ; (RNS): Reactive nitrogen species; (ROS): Reactive oxygen species; (SNpc): Subtantia nigra pars compacta; (TH): Tyrosine hydroxylase; (VM): Ventral midbrain; (WB): Western blot.

## Competing interests

The authors declare that they have no competing interests.

## Authors' contribution

FL participated in the design and Ms editing, in all treatment procedures, data elaboration, gene expression and behavioural studies. CT did the immunohistochemical procedures and confocal image analyses, carried out most of the histhological and sterological estimations and graphic representations. SC participated in all treatment procedures and tissues processing for western blot analyses and elaboration of the data. NT participated in all treatment procedures and daily control for animal foodintake, weights and toxicity control at sacrifice, and participate to immunohistochemical procedures and data elaboration. P-A Serra and MC Morale were responsible for protocol design and for analyses of striatal neurochemistry. F. Impagnatiello participated in protocol design and HCT1026 measurements. BM conceived the study and design, analysed the data and prepared the manuscript. All authors read, discussed and approved the final manuscript.

## References

[B1] Tedroff JM Functional consequences of dopaminergic degeneration in Parkinson's disease Adv Neurol 1999 80 67 70 10410704

[B2] Rascol O Payoux P Ory F Ferriera JJ Brefel-Courbon C Montastruc JL Limitations of current Parkinson's disease therapy Ann Neurol 2003 53 Suppl 3 S3 12 10.1002/ana.10513 12666094

[B3] Warner TT Schapira AHV Genetic and environmental factors in the cause of Parkinson's disease Ann Neurol 2003 53 S16 S25 10.1002/ana.10487 12666095

[B4] Hindle JV Ageing, neurodegeneration and Parkinson's disease Age Ageing 2010 39 156 161 10.1093/ageing/afp223 20051606

[B5] McGeer P McGeer E Inflammation and neurodegeneration in Parkinson's disease Parkinsonism Relat Disord 2004 10 S3 7 10.1016/j.parkreldis.2004.01.005 15109580

[B6] Chen H Zhang SM Herman MA Schwarzschild MA Willett WC Colditz GA Speizer FE Non steroidal anti-inflammatory drugs and the risk of Parkinson's disease Arch Neurol 2003 60 1059 64 10.1001/archneur.60.8.1059 12925360

[B7] Chen H Jacobs EJ Schwarzschild MA McCullough ML Calle EE Thun MJ Ascherio A Nonsteroidal antiinflammatory drug use and the risk forParkinson's disease Ann of Neurol 2005 58 963 967 10.1002/ana.20682 16240369

[B8] Morale MC Serra PA Delogu MR Migheli R Rocchitta G Tirolo C Caniglia S Testa N L'Episcopo F Gennuso F Scoto GM Barden N Miele E Desole MS Marchetti B Glucocorticoid receptor deficiency increases vulnerability of the nigrostriatal dopaminergic system: critical role of glial nitric oxide The FASEB Journal 2004 18 164 166 14630699 10.1096/fj.03-0501fje

[B9] Marchetti B Kettenmann H Streit WJ Glia-Neuron Crosstalk in Neuroinflammation, Neurodegeneration and Neuroprotection Brain Res Review Special Issue 2005 482 129 489

[B10] Marchetti B Serra PA L'Episcopo F Tirolo C Caniglia S Testa N Cioni S Gennuso F Rocchitta G Desole MS Mazzarino MC Miele E Morale MC Hormones are key actors in gene × environment interactions programming the vulnerability to Parkinson's disease: Glia as a common final pathway Ann NY Acad Sci 2005 1057 296 318 10.1196/annals.1356.023 16399902

[B11] Marchetti B Serra PA Tirolo C L'Episcopo F Caniglia S Gennuso F Testa N Miele E Desole MS Barden N Morale MC Glucocorticoid receptor-nitric oxide crosstalk and vulnerability to experimental Parkinsonism: pivotal role for glia-neuron interactions Brain Res Reviews 2005 48 302 321 10.1016/j.brainresrev.2004.12.030 15850669

[B12] Morale MC L'Episcopo F Tirolo C Giaquinta G Caniglia S Testa N Arcieri P Serra PA Lupo G Alberghina M Harada N Honda S Panzica GC Marchetti B Loss of Aromatase Cytochrome P450 function as a risk factor for Parkinson's disease? Brain Res Rev 2008 57 431 443 10.1016/j.brainresrev.2007.10.011 18063054

[B13] Hirsch EC Hunot S Neuroinflammation in Parkinson's disease: a target for neuroprotection? Lancet Neurol 2009 8 382 397 10.1016/S1474-4422(09)70062-6 19296921

[B14] Gao HM Hong JS Why neurodegenerative diseases are progressive: uncontrolled inflammation drives disease progression Trends Immunol 2008 29 357 65 10.1016/j.it.2008.05.002 18599350 PMC4794280

[B15] Przedborski S Inflammation and Parkinson's disease pathogenesis Mov disord 2010 25 Suppl 1 S55 7 10.1002/mds.22638 20187228

[B16] Hoang T Choi DK Nagai M Wu DC Nagata T Prou D Wilson GL Vila M Jackson-Lewis V Dawson V Dawson TM Chesselet MF Przedborski S Neuronal NOS and ciyclooxygenase-2 contribute to DNA damage in a mouse model of Parkinson's Disease Free Radical Biology & Medicine 2009 47 1049 1056 10.1016/j.freeradbiomed.2009.07.013 PMC3690578 19616617

[B17] Boger HA Granholm AC McGinty JF Middaugh LD A dual-hit animal model for age-related parkinsonism Prog in Neurobiol 2010 90 217 229 10.1016/j.pneurobio.2009.10.013 PMC3991553 19853012

[B18] L'Episcopo F Tirolo C Testa N Caniglia S Morale MC Marchetti B Glia as a turning point in the therapeutic strategy in Parkinson's disease CNS Neurol Disord Drug Targets 2010 9 349 72 20438439 10.2174/187152710791292639

[B19] McGeer PL Itagaki S Boyes BE McGeer EG Reactive microglia are positive for HLA-DR in the substantia nigra of Parkinson's and Alzheimer's disease brains Neurology 1988 38 1285 91 3399080 10.1212/wnl.38.8.1285

[B20] Langston JW Forno LS Tetrud J Reevers AG Kaplan JA Karluk D Evidence of active nerve cell degeneration in the substantia nigra of humans years after 1-methyl-4-phenyl-1,2,3,6-tetrahydropyridine exposure Ann Neurol 1999 46 598 605 10.1002/1531-8249(199910)46:4<598::AID-ANA7>3.0.CO;2-F 10514096

[B21] Kurkowska-Jastrzebska I Wrońska A Kohutnicka M Członkowski A Członkowska A The inflammatory reaction following 1-methyl-4-phenyl-1,2,3,6-tetrahydropyridine intoxication in mouse Exp Neurol 1999 156 50 61 10.1006/exnr.1998.6993 10192776

[B22] Gao HM Liu B Zhang W Hong JS Novel anti-inflammatory therapy for Parkinson's disease Trends Pharmacol Sci 2004 24 395 401 10.1016/S0165-6147(03)00176-7 12915048

[B23] McGeer P McGeer EG Glial reactions in Parkinson's disease Mov Disord 2008 23 474 83 10.1002/mds.21751 18044695

[B24] Marchetti B Abbracchio MP To be or not to be (inflamed) is that the question in anti-inflammatory drug therapy of neurodegenerative diseases? Trends in Pharmacological Sci 2005 26 517 525 10.1016/j.tips.2005.08.007 16126283

[B25] Whitton PS Inflammation as a causative factor in the aetiology of Parkinson's disease Brit J Pharmacol 2007 150 963 976 10.1038/sj.bjp.0707167 PMC2013918 17339843

[B26] Tansey MG Goldberg MS Neuroinflammation in Parkinson's disease: its role in neuronal death and implications for therapeutic intervention Neurobiol Dis 2010 37 510 8 10.1016/j.nbd.2009.11.004 19913097 PMC2823829

[B27] Liberatore GT Jacksons-Lewis V Vukosavic C Mandir AS Vila M McAuliffe WG Inducibile nitric oxide synthase stimulates dopaminergic neurodegeneration in the MPTP model of Parkinson's disease Nat Med 1999 5 1403 1409 10.1038/70978 10581083

[B28] Wu DC Jackson-Lewis V Vila M Tieu K Teismann P Vadseth C Blockade of microglial activation is neuroprotective in the 1-methyl-4-phenyl-1,2,3,6-tetrahydropyridine mouse model of Parkinson's disease J Neurosci 2002 22 1763 71 11880505 10.1523/JNEUROSCI.22-05-01763.2002 PMC6758858

[B29] Zhang W Wang T Pei Z Miller DS Wu X Blocl ML Wilson B Zhang W Zhou Y Hong JS Zhang J Aggragated alpha synuclein activates microglia: a process leading to disease progression in Parkinson's disease FASEB J 2005 19 533 542 10.1096/fj.04-2751com 15791003

[B30] Zhang W Dallas S Zhang D Guo JP Pang H Wilson B Microglial PHOX and Mac-1 are essential to the enhanced dopaminergic neurodegeneration elicited by A30P and A53T mutant Alpha-Synuclein Glia 2007 55 1178 1188 10.1002/glia.20532 17600340

[B31] Purisai MG McCormack AL Cumine S Li J Isla MZ Di Monte D Microglial activation is a priming event leading tom paraquat-induced dopaminergic cell degeneration Neurobiol Dis 2007 25 392 400 10.1016/j.nbd.2006.10.008 17166727 PMC2001246

[B32] Hu X Zhang D Pang H Caudle WM Li Y Gao H Macrophage antigen complex-1 mediates reactive microgliosis and progressive dopaminergic neurodegeneration in the MPTP model of Parkinson's disease J Immunol 2008 181 7194 7204 18981141 10.4049/jimmunol.181.10.7194 PMC2759089

[B33] Mander P Brown G Activation of microglial NADPH oxidase is synergistic with glial iNOS expression in inducing neuronal death: a dual key mechanism of inflammatory degeneration J Neuroinflammation 2005 2 20 27 10.1186/1742-2094-2-20 16156895 PMC1232863

[B34] Dehmer T Lindenau J Haid S Dichgans J Schulz JB Deficiency of inducible nitric oxide synthase protects against MPTP toxicity in vivo J Neurochem 2000 74 2213 6 10.1046/j.1471-4159.2000.0742213.x 10800968

[B35] Iravani MM Kashefi K Mander P Rose S Jenner P Involvement of inducible nitric oxide synthase in inflammation-induced dopaminergic neurodegeneration Neuroscience 2002 110 49 58 10.1016/S0306-4522(01)00562-0 11882372

[B36] Du Y Ma Z Lin S Dodel RC Gao F Bales K Triarhou LC Chernet E Perry KW Nelson DL Luecke S Phebus LA Bymaster FP Paul SM Minocycline prevents nigrostriatal dopaminergic neurodegeneration in MPTP model of Parkinson's disease Proc Natl Acad Sci 2001 98 14669 14674 10.1073/pnas.251341998 11724929 PMC64739

[B37] Teismann P Ferger B Inhibition of the cyclooxygenase isoenzymes COX-1 and COX-2 provide neuroprotection in the MPTP-mouse model of Parkinson's disease Synapse 2001 39 167 74 10.1002/1098-2396(200102)39:2<167::AID-SYN8>3.0.CO;2-U 11180504

[B38] Teismann P Tieu K Choi DK Wu DC Naini A Hunot S Vila M Jackson-Lewis V Przedborski S Cyclooxygenase-2 is instrumental in Parkinson's disease neurodegeneration Proc Natl Acad Sci 2003 100 5473 5478 10.1073/pnas.0837397100 12702778 PMC154369

[B39] Kurkowska-Jastrzebska I Babiuch M Joniec I Przybyłkowski A Członkowski A Członkowska A Indomethacin protects against neurodegeneration caused by MPTP intoxication in mice Int Immunopharmacol 2002 2 1213 8 10.1016/S1567-5769(02)00078-4 12349958

[B40] Kurkowska-Jastrzebska I Litwin T Joniec I Ciesielska A Przybyłkowski A Członkowski A Członkowska A Dexamethasone protects against dopaminergic neurons damage in a mouse model of Parkinson's disease Int Immunopharmacol 2004 4 1307 18 10.1016/j.intimp.2004.05.006 15313429

[B41] Kurkowska-Jjastrezba I Członkowski A Członkowska A Ibuprofen and the mouse model of Parkinson's disease Ann Neurol 2006 59 988 9 10.1002/ana.20860 16718707

[B42] Vijitruth R Liu M Choi DY Nguyen XB Hunter RL Bing G Ciclooxygenase-2 mediates microglial activation and secondary dopaminergic cell death in the mouse MPTP model Parkinson's disease J Neuroinflammation 2006 3 6 10.1186/1742-2094-3-6 16566823 PMC1440849

[B43] Sanchez-Pernaute R Ferree A Cooper O Yu M Brownell AL Isacson O Selective COX2 inhibition prevents progressive dopamine neuron degeneration in a rat model of Parkinson's disease J Neuroinflammation 2004 1 6 16 10.1186/1742-2094-1-6 15285796 PMC483059

[B44] Sairam K Saravanan KS Banerjee R Mohanakumar KP Non-steroidal anti-inflammatory drug sodium salicylate, but not diclofenac or celecoxib, protects against 1-methyl-4-phenyl pyridinium-induced dopaminergic neurotoxicity in rats Brain Res 2003 966 245 52 10.1016/S0006-8993(02)04174-4 12618347

[B45] Esposito E Di Matteo V Benigno A Pierucci M Crescimanno G Di Giovanni G Non-steroidal anti-inflammatory drugs in Parkinson's disease Exp Neurology 2007 205 295 312 10.1016/j.expneurol.2007.02.008 17433296

[B46] Asanuma M Miyazaki I Common anti-inflammatory drugs are potentially therapeutic for Parkinson's disease? Exp Neurology 2007 206 172 178 10.1016/j.expneurol.2007.05.006 17599833

[B47] Asanuma M Miyazaki I Non-steroidal anti-inflammatory drugs in Parkinson's disease: possible involvement of quinone formation Expert Rev Neurotherapeutics 2006 6 1313 1325 10.1586/14737175.6.9.1313 17009919

[B48] Asanuma M Miyazaki I Kohno M Ogawa N Neuroprotective effects on non-steroidal anti-inflammatory drugs by direct scavenging of nitric oxide radicals J Neurochem 2001 76 1895 1904 10.1046/j.1471-4159.2001.00205.x 11259508

[B49] Samii A Etminan M Wiens MO Jafari S NSAID use and the risk of Parkinson's disease: systematic review and meta-analysis of observational studies Drugs Aging 2009 26 769 779 10.2165/11316780-000000000-00000 19728750

[B50] Gagne JJ Power MC Anti-inflammatory drugs and risk of Parkinson's disease: a meta-analysis Neurology 2010 74 995 1002 10.1212/WNL.0b013e3181d5a4a3 20308684 PMC2848103

[B51] Klegeris A McGeer PL Non-steroidal anti-inflammatory drugs (NSAIDs) and other anti-inflammatory agents in the treatment of neurodegenerative disease Curr Alzheimer Res 2005 2 355 65 10.2174/1567205054367883 15974901

[B52] Klegeris A McGeer EG McGeer PL Therapeutic approaches to inflammation in neurodegenerative disease Curr Opin Neurol 2007 20 351 7 10.1097/WCO.0b013e3280adc943 17495632

[B53] Minghetti L Cyclooxygenase-2 (COX-2) in inflammatory and degenerative brain diseases J Neuropathol Exp Neurol 2004 63 901 10 15453089 10.1093/jnen/63.9.901

[B54] Patrignani P Tacconelli S Sciulli MG Capone ML New insights into COX-2 biology and inhibition Brain Res Brain Res Rev 2005 48 352 9 10.1016/j.brainresrev.2004.12.024 15850674

[B55] Needleman P Isakson PC The discovery and function of COX-2 J Rheumatol Suppl 1997 49 6 8 9249644

[B56] Rao P Knaus EE Evolution of nonsteroidal anti-inflammatory drugs (NSAIDs): cyclooxygenase (COX) inhibition and beyond J Pharm Pharm Sci 2008 11 81s 110s 19203472 10.18433/j3t886

[B57] Wallace B Reuter C Cicala W McKnight MB Grisham G Cirino A Diclofenac derivative without ulcerogenic properties J Pharmacol 1994 257 249 255 10.1016/0014-2999(94)90136-8 8088345

[B58] Wallace JL Ignarro LJ Fiorucci S Potential cardioprotective actions of NO releasing aspirin Nat Rev Drug Disc 2002 1 375 382 10.1038/nrd794 12120413

[B59] Fiorucci S Antonelli E Santucci L Morelli O Miglietti M Federici B Gastrointestinal safety of nitric oxide-derived aspirin is related to inhibition of ICE-like cysteine proteases in rats Gastroenterology 1999 116 1089 1106 10.1016/S0016-5085(99)70012-0 10220501

[B60] Fiorucci S Antonelli E NO-NSAIDs: from inflammatory mediators to clinical readouts Inflamm Allergy Drug Targets 2006 5 121 131 10.2174/187152806776383161 16613571

[B61] Fiorucci S Santucci L Distrutti E NSAIDs, coxibs, CINOD and H_2_S-releasingNSAIDs: what lies beyond the horizon Digest and Liv Dis 2007 39 1043 1051 10.1016/j.dld.2007.09.001 17997373

[B62] Ajmone-Cat MA Nicolini A Minghetti L Differential effects of the nonsteroidal antiinflammatory drug flurbiprofen and nitric oxide-releasing derivative, nitroflurbiprofen, on prostaglandin E(2), interleukin-1beta, and nitric oxide synthesis by activated microglia J Neurosci Res 2001 66 715 722 10.1002/jnr.10038 11746392

[B63] Bernardo A Ajmone-Cat MA Gasparini L Ongini E Minghetti L Nuclear receptor peroxisome proliferator-activated receptor-gamma is activated in rat microglial cells by the anti-inflammatory drug HCT1026, a derivative of flurbiprofen J Neurochem 2005 92 895 903 10.1111/j.1471-4159.2004.02932.x 15686492

[B64] Idris Al Ralston SH van't Hof RJ The nitrosylated flurbiprofen derivative HCT1026 inhibits cytokine-induced signalling through a novel mechanism of action Eur J Pharmacol 2009 602 215 222 10.1016/j.ejphar.2008.11.023 19046964

[B65] Keeble JE Moore PK Pharmacology and potential therapeutic applications of nitric oxide-releasing non-steroidal anti-inflammatory and related nitric oxide-donating drugs Br J Pharmacol 2002 137 295 340 10.1038/sj.bjp.0704876 12237248 PMC1573498

[B66] Furlan R Kurne A Bergami A Brambilla E Maucci R Gasparini L Butti E Comi G Ongini E Martino G A nitric oxide releasing derivative of flurbiprofen inhibits experimental autoimmune encephalomyelitis J Neuroimmunol 2004 150 10 9 10.1016/j.jneuroim.2004.01.004 15081244

[B67] Jackson-Lewis V Jakowec M Burke RE Przedborski S Time course and morphology of dopaminergic neuronal death caused by the neurotoxin 1-methyl-4-phenyl-1,2,3,6 tetrahydropyridine Neurodegeneration 1995 4 257 69 10.1016/1055-8330(95)90015-2 8581558

[B68] Tatton NA Kish SJ In situ detection of apoptotic nuclei in the substantia nigra compacta of 1-methyl-4-phenyl-1,2,3,6-tetrahydropyridine-treated mice using terminal deoxynucleotidyl transferase labelling and acridine orange staining Neuroscience 1997 77 1037 48 10.1016/S0306-4522(96)00545-3 9130785

[B69] Jackson-Lewis V Przedborski S Protocol for the MPTP model of Parkinson's disease Nature Protocols 2007 2 141 151 10.1038/nprot.2006.342 17401348

[B70] Aldini G Carini M Orioli M Maffei Facino R Wenk GL Metabolic profile of NO-flurbiprofen (HCT1026) in rat brain and plasma: an LC-MS study Life Sci 2002 71 1487 1500 10.1016/S0024-3205(02)01915-X 12127904

[B71] Lowry OH Rosebrough NJ Farr AL Randall RJ Protein measurement with the Folin phenol reagent J Biol Chem 1951 193 265 75 14907713

[B72] Franklin KBJ Paxinos G The mouse brain in stereotaxic coordinates Academic Press Inc 1997

[B73] Baquet ZC Williams D Brody J Smeyne RJ A comparison of model-basaed (2D) and design-based (3D) stereological methods for estimating cell number in the subtantia nigra pars compacta (SNpc) of the C57BL/6J mouse Neuroscience 2009 161 1082 1090 10.1016/j.neuroscience.2009.04.031 19376196 PMC2705113

[B74] Abercrombie M Estimation of nuclear population from microtome sections Anat Rec 1946 94 239 247 10.1002/ar.1090940210 21015608

[B75] Kreutzberg GW Microglia: a sensor for pathological events in the CNS Trends Neurosci 1996 19 312 8 10.1016/0166-2236(96)10049-7 8843599

[B76] Gainetdinov RR Fumagalli F Joness SR Caron MG Dopamine transporter is required for in vivo MPTP neurotoxicity: evidence from mice lacking the transporter J Neurochem 1997 69 1322 1325 10.1046/j.1471-4159.1997.69031322.x 9282960

[B77] Ho A Blum M Induction of interleukin-1 associated with compensatory dopaminergic sprouting in the denervated striatum of young mice: model of aging and neurodegenerative disease J Neurosci 1998 18 5614 29 9671653 10.1523/JNEUROSCI.18-15-05614.1998 PMC6793059

[B78] Date I Felten DL Felten SY Long-term effect of MPTP in the mouse brain in relation to aging: neurochemical and immunocytochemical analysis Brain Res 1990 519 266 76 10.1016/0006-8993(90)90088-S 1975765

[B79] Sedelis M Schwarting RKW Husto JP Behavioral phenotyping of the MPTP mouse model of Parkinson's disease Behav Brain Res 2001 125 109 122 10.1016/S0166-4328(01)00309-6 11682102

[B80] Alvarez-Fisher D Henze C Strenzke C Westrich J Ferger B Holinger GU Oertel WH Hartmann A Characterization of the striatal 6-OHDA model of Parkinson's disease in wild type and α-synuclein-deleted mice Exp Neurol 2007 210 182 193 10.1016/j.expneurol.2007.10.012 18053987

[B81] Pott Godoy MC Tarelli R Ferrari CC Sarchi MI Pitossi FJ Central and systemic IL-1 exacerbates neurodegeneration and motor symptoms in a model of Parkinson's disease Brain 2008 131 1880 94 10.1093/brain/awn101 18504291 PMC2442423

[B82] Hunter RL Cheng B Choi DY Liu M Liu S Cass WA Intrastriatal lipopolysaccharide injection induces parkinsonism in C57/B6 mice J Neurosci Res 2009 87 1913 1921 10.1002/jnr.22012 19224579 PMC2692550

[B83] Pierre SR Lemmens MAM Figueiredo-Pereira ME Subchronic infusion of the product of inflammation prostaglandin J2 models sporadic Parkinson's disease in mice J of Neuroinflammation 2009 6 18 10.1186/1742-2094-6-18 PMC2724408 19630993

[B84] Moncada S Nitric oxide in the vasculature: physiology and pathophysiology Ann N Y Acad Sci 1997 811 60 67 10.1111/j.1749-6632.1997.tb51989.x 9186585

[B85] Ritter JM Harding I Warren JB Precaution, cyclooxygenase inhibition and cardiovascular risk Trends in Pharmacol Sci 2009 30 503 508 10.1016/j.tips.2009.07.007 19762092

[B86] Prosperi C Scali C Pepeu G Casamenti F NO-flurbiprofen attenuates excitotoxin-induced brain inflammation, and releases nitric oxide in the brain Jpn J Pharmacol 2001 86 230 5 10.1254/jjp.86.230 11459126

[B87] Abdul-Hay SO Luo J Ashghodom RT Thatcher GRJ NO-flurbiprofen reduces amyloid β, is neuroprotective in cell culture, and anhances cognition in responce to cholinergic blockade J Neurochem 2009 111 766 776 10.1111/j.1471-4159.2009.06353.x 19702655 PMC2792633

[B88] Bezard E Jaber M Gonon F Boireau A Bloch B Gross CE Adaptive changes in the nigrostriatal pathway in response to increased 1-methyl-4-phenyl-1,2,3,6-tetrahydropyridine-induced neurodegeneration in the mouse Eur J Neurosci 2000 8 2892 900 10.1046/j.1460-9568.2000.00180.x 10971632

[B89] Collier TJ Lipton J Dalkey BF Palfi S Chu Y Sortwell C Bakai RAE Sladek JR Jr Kordower JJH Aging related changes in the nigrostriatal dopamine system and the response to MPTP in nonhuman primates: diminished compensatory mechanisms as a prelude to parkinsonism Neurobiol Dis 2007 26 56 65 10.1016/j.nbd.2006.11.013 17254792 PMC1899875

[B90] Przybylkowski A Przybylkowski A Kurkowska-Jastrzebska I Cyclooxygenases mRNA and protein expression in striata in the experimental mouse model of Parkinson's disease induced by 1-methyl-4-phenyl-1,2,3,6-tetrahydropyridine administration to mouse Brain Res 2004 1019 144 151 10.1016/j.brainres.2004.05.095 15306248

[B91] Kaanan NM Kordower JH Collier TJ Age-related changes in dopamine transporters and accumulation of 3-nitrotyrosine in rhesus monkey midbrain dopamine neurons: relevance in selective neuronal vulnerability to degeneration Eur J Neurosci 2008 27 3205 3215 10.1111/j.1460-9568.2008.06307.x 18598263 PMC3391583

[B92] Gasparini L Ongini E Wilcock D Morgan D Activity of flurbiprofen and chemically related anti-inflammatory drugs in models of Alzheimer's disease Brain Res Brain Res Rev 2005 48 400 8 10.1016/j.brainresrev.2004.12.029 15850679

[B93] Brunelli S Sciorati C D'Antona G Innocenzi A Covarello D Perrotta C Monopoli A Sanvito F Bottinelli R Ongini E Cossu G Clementi E Nitric oxide release combined with non-steroisal anti-inflammatory activity prevents muscular distrophy pathology and enhances stem cell therapy Proc Natl Acad Sci 2007 104 264 269 10.1073/pnas.0608277104 17182743 PMC1765447

[B94] Clancy R Varenika B Huang W Ballou L Attur M Amin AR Abramson SB Nitric oxide synthase/COX cross-talk: nitric oxide activates COX-1 but inhibits COX-2-derived prostaglandin production J Immunol 2000 165 1582 7 10903767 10.4049/jimmunol.165.3.1582

[B95] Colasanti M Suzuki K The dual personality of NO Trends Pharmacol Sci 2000 21 249 252 10.1016/S0165-6147(00)01499-1 10979862

[B96] Guastadisegni C Minghetti L Nicolini A Polazzi E Ade P Balduzzi M Levi G Prostaglandin E2 synthesis is differentially affected by reactive nitrogen intermediates in cultured rat microglia and RAW 264.7 cells FEBS Lett 1997 413 314 8 10.1016/S0014-5793(97)00925-3 9280304

[B97] Guastadisegni C Nicolini A Balduzzi M Ajmone-Cat MA Minghetti L Modulation of PGE(2) and TNFalpha by nitric oxide and LPS-activated RAW 264.7 cells Cytokine 2002 19 175 80 10.1006/cyto.2002.1955 12297110

[B98] Rocchitta G Migheli R Mura MP Grella G Esposito G Marchetti B Miele E Desole MS Miele M Serra P-A Signaling pathways in the nitric oxide and iron-induced dopamine release in the striatum of freely mooving rats: role of extracellular Ca2+ and L-type Ca2+ channels Brain Res 2005 1047 18 29 10.1016/j.brainres.2005.04.008 15890318

[B99] Sugama S Yang L Cho BP DeGiorgio LA Lorenzl S Albers DS Beal MF Volpe BT Joh TH Age-related microglial activation in 1-methyl-4-phenyl-1,2,3,6-tetrahydropyridine (MPTP)-induced dopaminergic neurodegeneration in C57BL/6 mice Brain Res 2003 964 288 94 10.1016/S0006-8993(02)04085-4 12576189

[B100] Sawada H Hishida R Hirata Y Ono K Suzuki H Muramatsu S Nakano I Nagatsu T Sawada M Activated microglia affect the nigro-striatal dopamine neurons differently in neonatal and aged mice treated with 1-Methyl-4-Phenyl-1,2,3,6-Tetrahydropyridine J Neurosci Res 2007 85 1752 1761 10.1002/jnr.21241 17469135

[B101] Streit WJ Mrak RE Griffin WS Microglia and neuroinflammation: a pathological perspective J Neuroinflammation 2004 1 14 10.1186/1742-2094-1-14 15285801 PMC509427

[B102] Lucin KM Wyss-Coray T Immune activation in brain aging and neurodegeneration: too much or too little? Neuron 2009 64 110 122 10.1016/j.neuron.2009.08.039 19840553 PMC2834890

[B103] Sandhu JK Gardaneh M Iwasiow R Lanthier P Gangaraju S Ribecco-Lutkiewicz M Astrocyte-secreted GDNF and glutathione antioxidant system protect neurons against 6OHDA cytotoxicity Neurobiology of Disease 2009 33 405 414 10.1016/j.nbd.2008.11.016 19118631

[B104] Smeyne M Goloubeva O Smeyne RJ Strain-dependent susceptibility to MPTP and MPTP(+)-induced parkinsonism is determined by glia Glia 2001 34 73 80 10.1002/glia.1042 11307156

